# Pickering Emulsion-Driven MXene/Silk Fibroin Hydrogels with Programmable Functional Networks for EMI Shielding and Solar Evaporation

**DOI:** 10.1007/s40820-025-01818-w

**Published:** 2025-06-24

**Authors:** Guang Yin, Jing Wu, Chengzhang Qi, Xinfeng Zhou, Zhong-Zhen Yu, Hao-Bin Zhang

**Affiliations:** 1 State Key Laboratory of Organic-Inorganic Composites, Beijing University of Chemical Technology, Beijing, 100029 People’s Republic of China; 2 Center for Nanomaterials and Nanocomposites, College of Materials Science and Engineering, Beijing University of Chemical Technology, Beijing, 100029 People’s Republic of China

**Keywords:** MXene, Silk fibroin, Pickering emulsion, Electromagnetic interference shielding, Solar-driven evaporation

## Abstract

Flexible and conformable nanomaterial-based functional hydrogels find promising applications in various fields. However, the controllable manipulation of functional electron/mass transport networks in hydrogels remains rather challenging to realize. We describe a general and versatile surfactant-free emulsion construction strategy to customize robust functional hydrogels with programmable hierarchical structures. Significantly, the amphipathy of silk fibroin (SF) and the reinforcement effect of MXene nanosheets produce sable Pickering emulsion without any surfactant. The followed microphase separation and self-cross-linking of the SF chains induced by the solvent exchange convert the composite emulsions into high-performance hydrogels with tunable microstructures and functionalities. As a proof-of-concept, the controllable regulation of the ordered conductive network and the water polarization effect confer the hydrogels with an intriguing electromagnetic interference shielding efficiency (~ 64 dB). Also, the microstructures of functional hydrogels are modulated to promote mass/heat transfer properties. The amino acids of SF and the surface terminations of MXene help reduce the enthalpy of water evaporation and the hierarchical structures of the hydrogels accelerate evaporation process, expecting far superior evaporation performance (~ 3.5 kg m⁻^2^ h⁻^1^) and salt tolerance capability compared to other hydrogel evaporators. Our findings open a wealth of opportunities for producing functional hydrogel devices with integrated structure-dependent properties.

**Supplementary Information:**

The online version contains supplementary material available at 10.1007/s40820-025-01818-w.

## Highlights

- A versatile surfactant-free emulsion construction strategy is proposed to customize functional hydrogels.
- The synergistic emulsification mechanism between amphiphilic polymers and MXene is comprehensively elucidated.
- Programmable functional structures confer hydrogels with excellent EMI shielding and water evaporation performance.

**Supplementary Information:**

The online version contains supplementary material available at 10.1007/s40820-025-01818-w.

## Graphical abstract

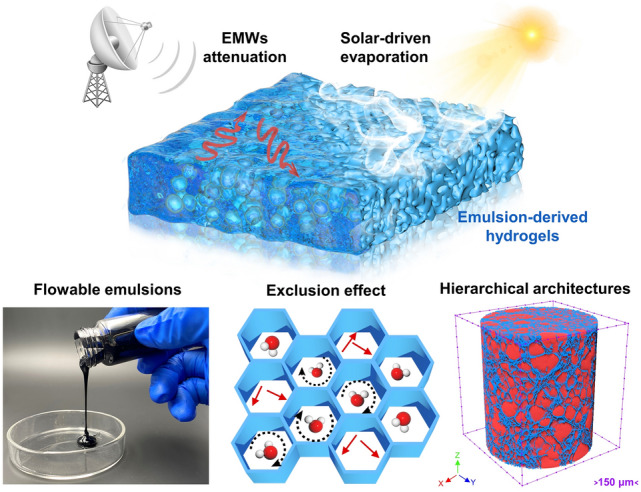

## Introduction

Flexible and robust functional hydrogels find widespread applications in soft robotics [1], flexible electronics [2], electromagnetic radiation protection [3], and energy conversion [4]. Beyond conventional ionic and conductive polymer-based hydrogels, various composite hydrogels produced by including different conductive nanomaterials, such as graphene [5], MXene [6], carbon nanotubes [7], and metallic nanomaterials [8], offer more newfangled attributes and promising candidates for different applications. Fundamentally, the functionality realization of these nanomaterials depends on the type of nanofillers, their distribution in the matrix, and the microstructures of the hydrogel themselves. Recently, researchers make great efforts to address the interfacial incompatibility between conductive fillers and polymer matrices, which severely impedes the uniform dispersion, and modulation of hierarchical architectures in the hydrogels. For example, some attempts adopt directional freezing [9], sacrificial templates [10], and cononsolvency strategies [11] to form directional channels and anisotropic pores in the composite hydrogels. However, these methods largely change the structures of hydrogels by additional procedures (e.g., ice crystal template and microphere template etching) and neglect the effect of the microstructures of hydrogel components, limiting the flexible regulation of functionality for hydrogels. Importantly, although massive randomly dispersed nanomaterials can form functional structures in the composite hydrogels, severe agglomeration and early gelation phenomena inevitably deteriorate their comprehensive performance [12, 13].

Inspired by the exclusion effect in immiscible polymer blends, it is possible to modulate the phase structures and filler distribution for constructing functional structures [14–16]. Similarly, Pickering emulsions, consisting of a tunable dispersed phase and a continuous phase, offer a feasible and versatile platform for the structure design and performance regulation of hydrogels. For example, the interfacial assembly of nanomaterials at the two-phase interfaces, rather than random dispersion in individual phases of emulsions, can enable almost all nanomaterials to construct connected architectures, resembling the select distribution of fillers in the polymer composites [17]. Significantly, the micro-structure characteristics, including phase structure, continuity, and distribution, of the derived functional hydrogels can be freely tuned on-demand for specific applications. Despite the promising prospects, some critical issues should be well addressed to develop functional hydrogels from Pickering emulsions. Firstly, proper chemical/physical cross-linking is crucial for the transformation from flowable emulsion to robust hydrogel, yet too vigorous polymerization or cross-linking reactions will induce a demulsification effect. Secondly, stable and robust two-phase interfaces are required to prevent breakage and coalescence of emulsion droplets with the assistance of various ligands. Finally, the different charges, polarity and interfacial affinity between surfactants and components may cause filler agglomeration or premature gelation of emulsions. Therefore, some general yet effective strategies are required to develop robust functional hydrogels with precisely customized hierarchical structures for potential applications.

Here, we propose a versatile surfactant-free emulsion construction strategy to produce functional hydrogels with tunable structures and functionalities by exploiting the amphipathy of silk fibroin (SF) chains and the synergistic reinforcement effect of MXene nanosheets. The followed microphase separation and self-cross-linking of SF chains induced by the solvent exchange convert the emulsions into high-performance functional hydrogels. The surfactant-free emulsification mechanism for the formation of the robust two-phase interfaces is explored by theoretical calculations and dynamic simulations. The emulsion-derived hydrogels exhibit outstanding structural stability and processability than non-emulsified hydrogels owing to the exclusion effect and strong interfaces. As a proof-of-concept, the optimized conductive network and the water polarization effect confer the hydrogels with intriguing electromagnetic interference (EMI) shielding performance (~ 64 dB). The variable emulsion structures dominate the porous structures for oil-in-water (O/W) hydrogels, which allow for the free regulation of the water evaporation rate. The obtained evaporation performance (~ 3.5 kg m⁻^2^ h⁻^1^) and salt tolerance capability are obviously superior to other hydrogel evaporators.

## Experimental Section

### Materials

Bombyx mori (B. mori) silkworm cocoons and Ti_3_AlC_2_ MAX powder (400 mesh) were purchased from Shanxi Cansang Co. Ltd., China and FoShan XinXi Technology Co., Ltd., respectively. Polydiallyl dimethyl ammonium (PDDA), carboxylated multiwalled carbon nanotubes (CNTs, 8–15 nm), poly(3,4-ethylenedioxythiophene):poly(styrenesulfonate) (PEDOT:PSS), Lithium fluoride (LiF), lithium chloride (LiCl), calcium chloride (CaCl_2_), and sodium bicarbonate (NaHCO_3_) were supplied by Aladdin (China). Hydrochloric acid (HCl), formic acid (FA), and toluene were purchased from Beijing Chemical Reagents Co. Ltd. Silkworm cocoons were cut into small pieces and degummed in boiling water with 0.5 wt% NaHCO_3_ solution. The degummed SF fibrils were dissolved in the LiCl-FA system to prepare the SF/LiCl-FA solution (5%, w/v). Ti_3_C_2_T_*x*_ MXene nanosheets were synthesized by LiF/HCl solution etching of Ti_3_AlC_2_ MAX powder using previously reported method [6]. To improve the dispersion of MXene in LiCl-FA, PDDA solution (2 mg mL⁻^1^) was added to a specific MXene suspension (40 mg mL⁻^1^) and stirred for 3 h to obtain the positively charged PDDA-MXene (P-MXene) slurry (30 mg mL⁻^1^).

### Preparation of Composite Hydrogels

The P-MXene slurry and CNTs were slowly dispersed in SF/LiCl-FA solution. Unless particularly stated, the mass ratios of MXene to CNTs and SF were 1:4 and 1:10, respectively. Then, the oil phase (toluene) was added into the SF/CNTs/MXene (SCM) dispersion and uniformly mixed for 5 min (3500 r min^−1^) to obtain the SCM Pickering emulsion. Specifically, the oil phase worked as the dispersed phase in Pickering emulsion, whose volume fraction (*φ*) ranged from 0.17 to 0.40. Notably, the volume fraction exceeding 0.4 will cause severe demulsification effect and prevent the formation of homogeneous emulsion. The prepared emulsions were poured into the polytetrafluoroethylene molds and then placed them into the poor solvent (such as water) of SF for 24 h to solvent exchange, resulting in O/W hydrogels. The frozen SCM_(O/W)_ hydrogels were freeze-dried at − 60 °C and 10 Pa for 48 h to obtain the SCM aerogels. Similarly, the oil-in-water SF/MXene (SM_O/W_) hydrogels were prepared using the same procedure without adding CNTs.

### Characterizations

Morphology and micro-structure were observed with Hitachi S7800 field‑emission scanning electron microscope (SEM), Tecnai G2 F20 STWIN transmission electron microscope (TEM), and Nikon Eclipse 80i polarized optical microscope (POM). Energy dispersive X-ray spectrometry (EDS) mapping (Bruker EDX system) was used to obtain the distribution of main components in the hydrogel. 3D microstructures of porous aerogels were observed and recorded by the micro-CT (Bruker Skyscan1272). Fourier-transform infrared (FTIR) spectroscopy curves were recorded via a ThermoFisher Nicolet 8700 FTIR spectrometer. Rheological properties were measured using a flat plate rheometer (TA Instruments DHR-3). Mechanical date was obtained from a SUNS UTM4502XH tensile tester. Simulated solar light was derived from a CEL-HXUV300 solar simulator. Finite element analysis of electromagnetic performance was simulated by ANSYS HFSS 15.0 software.

## Results and Discussion

### Structure–Property Regulation of Composite Hydrogels

**Fig. 1:**
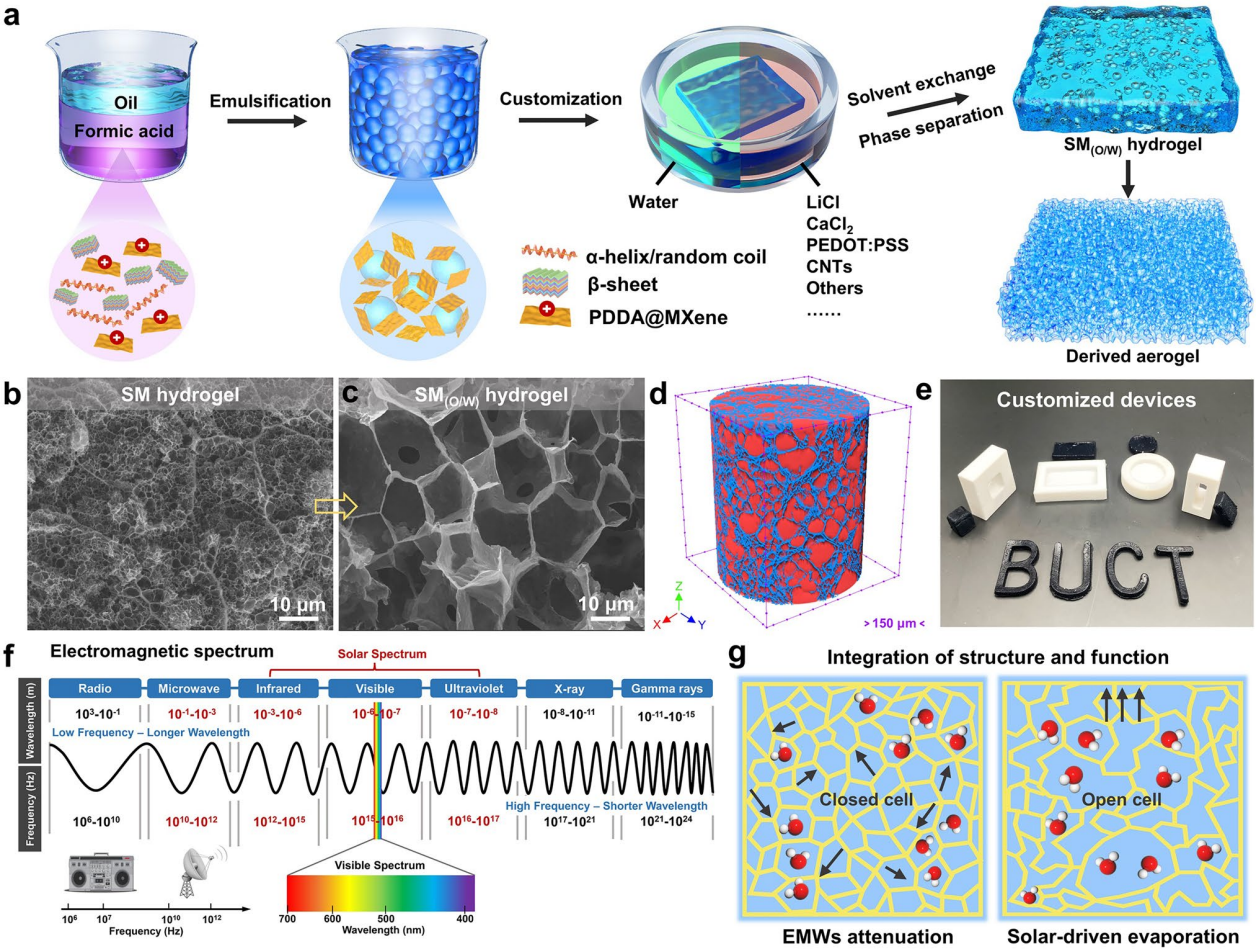
**a** Schematic illustrating the fabrication process of SM_(O/W)_ and its derived aerogels. SEM images of **b** SM and **c** SM_(O/W)_ hydrogels. **d** Three-dimensional (3D) reconstructed structure of the derived SM_(O/W)_ aerogel via micro-CT technique, the blue and red colors indicate the skeleton and pore, respectively. **e** Digital photograph of the customized SM_(O/W)_ hydrogels. **f** Applied microwave and visible bands of electromagnetic spectrum, and **g** the performance (EMI shielding and solar-thermal conversion) modulation mechanism dominated by the internal structure of SM_(O/W)_ hydrogels

To address the agglomeration behavior of nanomaterials inside composite hydrogels and to break through the stiff property regulation brought about by polymerization networks, we propose a one-step route to confer hydrogels with tunable functionalities based on the Pickering emulsions and microphase separation strategy (Fig. 1a). The amphiphilic polymers (e.g., gelatin, silk fibroin, and chitosan) can form homogeneous emulsions without surfactants in some oil components (e.g., toluene and cyclohexane), and the physical cross-linking or entanglement of macromolecular chains helps to construct hydrogel networks [18–20]. Initially, SF is dissolved in LiCl-FA solution (5%, w/v) and the positively charged MXene nanosheets modified with PDDA are introduced to form an aqueous mixture (Fig. S1). Then, the SF/MXene (SM) Pickering emulsion is formed with the existence of an oil phase (toluene) under homogenization shear without any surfactant. The amphiphilic SF chains and two-dimensional (2D) MXene nanosheets synergistically ensures the construction of stable two-phase interfaces. The obtained SM emulsion is casted in molds soaked in water for solvent exchange, which induces the microphase separation effect to produce O/W hydrogels (Fig. S2). The solubility discrepancy of SF chains in FA and water is responsible for the transformation of SF chains from aggregation, collapse, and physical cross-linking to micelles, microspheres, and finally to hydrogels [21–23]. Significantly, the required functionalities can be endowed to the hydrogels by adding different hydrosoluble materials (e.g., LiCl, CaCl_2_, PEDOT:PSS, and CNTs) during the solvent exchange process. The resulting SM_(O/W)_ hydrogels can be selectively converted into functional aerogels by freeze-drying treatment (Fig. 1a).

In contrast, non-emulsified SM hydrogels are prepared by blending SF and MXene and the following microphase separation process. The random distribution of constituents in the hydrogel yields numerous small pores (average diameter, ~ 1 μm) upon microphase separation and physical cross-linking (Fig. 1b). As the case of blending hydrogel, it is difficult to regulate functional pathways in the hydrogel at low filler loadings, while excess fillers easily cause possible agglomeration and uncontrollable gelation during the blending process. Inspiringly, the emulsion particles in the SM_(O/W)_ hydrogel can expel conductive nanofillers to form high-quality porous structures consisting of well-connected and tightly stacked struts and walls (~ 10 μm), resembling the volume exclusion effect reported for conductive polymer composites. All the nanomaterials are collected to assemble the conductive network and the isolated agglomeration is avoided. The regulated porous structure exhibits more efficient conductance and significantly reduces the percolation threshold of the hydrogels (Fig. 1c). At the same time, numerous smaller pores are observed in the cell walls because of the microphase separation effect, confirming the cross-linking of SF chains in Pickering emulsion (Fig. S3). Moreover, the volume fraction of the oil phase (*φ*) also greatly influences the hierarchical structures and the electron/mass transfer properties of SM_(O/W)_ hydrogels. Thus, the synergistic combination of Pickering emulsion and microphase separation propose a flexible yet versatile method to freely customize functional hydrogels for various specific applications (Fig. 1e), far superior over traditional strategies (e.g., directional freezing and sacrificial template).

Then, the removal of oil droplets from the SM_(O/W)_ hydrogels leaves similar ordered structures, and the robust and strong frameworks are formed with the increasing of SF content (Fig. S4). To explore the structure tunability, 5% SF and different volume fractions of the oil phase are adopted to prepare functional SM_(O/W)_ hydrogels. Clearly, the hydrogel shows intact and continuous pore structure (closed-cell) at *φ* = 0.2 (Fig. S5a), but partially fractured pores (open-cell) occur at *φ* = 0.4 owing to the excess of emulsion droplets (Fig. S5b). The close-celled structure can confer optimal conductive pathways, whereas the irregular open-celled structure may impair the overall architecture integrity and allow oil phase leakage. Interestingly, the open channels accelerate mass and heat transfer through the hydrogel for efficient energy conversion. Micro-CT image reveals the overall interior cellular structures of the SM_(O/W)_ hydrogel (*φ* = 0.4) and its derived aerogel (Fig. 1d). It is reasonably inferred that the incomplete skeleton (blue color) and uneven pores (red color) derive from the coalesce and fusion of oil droplets, as evidenced by the coexistence of large and small pores in SM_(O/W)_ hydrogel (Fig. S6). This structure–property regulatory mechanism induced by dynamic Pickering emulsion applies to energy dissipation and utilization in specific bands (microwave, infrared, visible, and ultraviolet bands) of the electromagnetic spectrum (Fig. 1f). In specific, in the microwave band, the attenuation of electromagnetic waves (EMWs) by conductive hydrogels mainly relies on the intact conductive skeleton and the efficient electron transport pathways. Compared to the SM hydrogel, the SM_(O/W)_ hydrogel with closed-celled conductive network greatly reinforces the attenuation of EMWs via the increased reflected interfaces (Fig. 1g). For the solar spectrum (infrared, visible, and ultraviolet bands), the input energy can be widely harvested, transformed, and utilized by SM_(O/W)_ hydrogels for water evaporation to solve water scarcity and pollution problems. The emulsion template of SM_(O/W)_ hydrogels provides larger open-celled edited="true" ID="null">hierarchical pore structure (large and small pores) help to establish efficient water transport channels, achieving excellent water evaporation rate and efficiency (Fig. 1g). More importantly, the EMI shielding and solar-driven evaporation properties are dynamically regulated with the volume fraction of dispersed phase (oil phase) in the Pickering emulsion. Therefore, the proposed route offers a helpful inspiration for developing tunable EMI shielding and efficient energy conversion hydrogel devices.

### Synergistic Emulsification Mechanism of SF and MXene

**Fig. 2:**
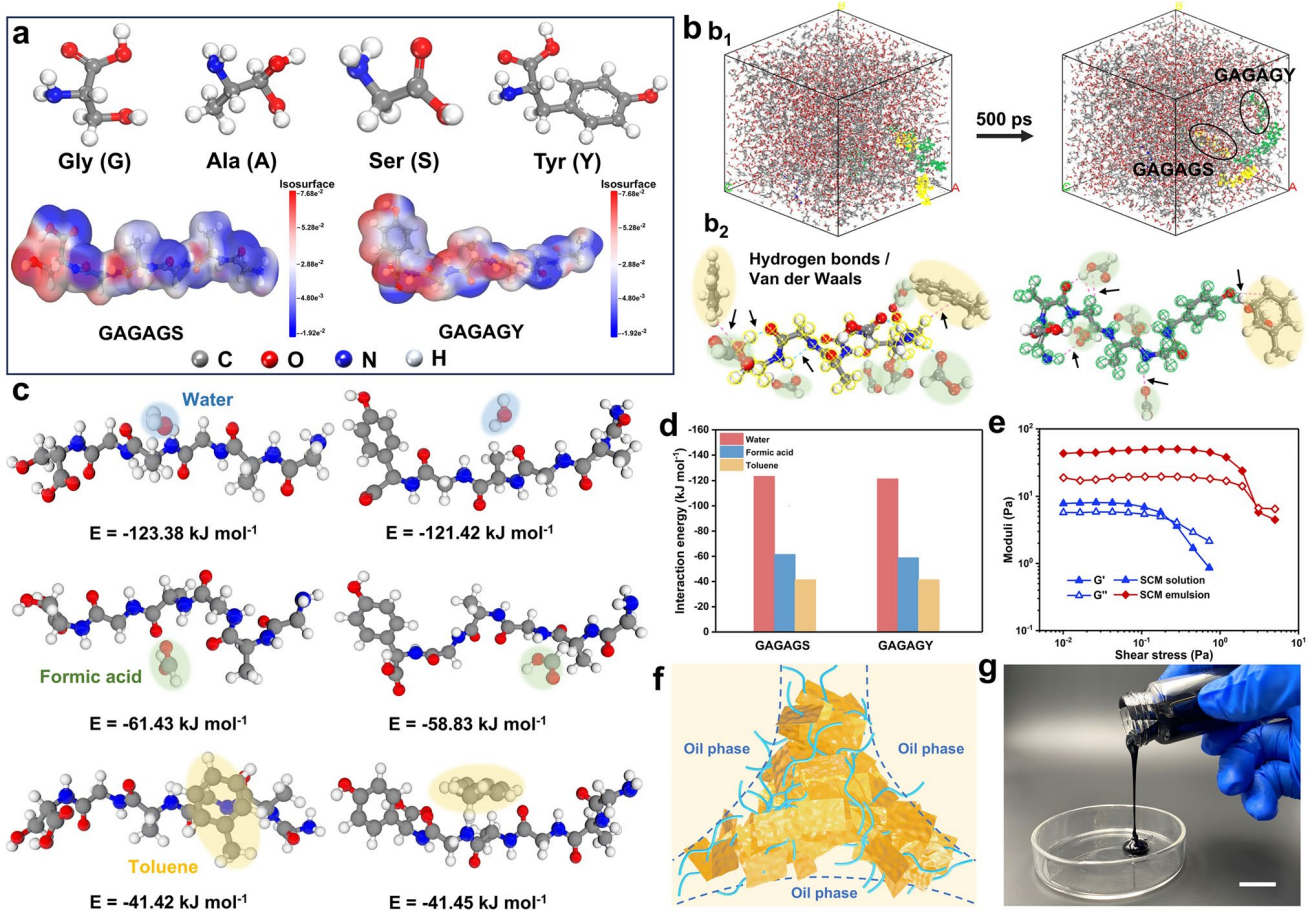
**a** Electrostatic potentials of GAGAGS and GAGAGY. **b** Molecular dynamics simulation of the SF-toluene unit at 0 and 500 ps snapshots. The yellow and green colors represent GAGAGS and GAGAGY, respectively.** c, d** Combined energies of SF sequences with different solvents (water, formic acid, and toluene) obtained by DFT calculation. **e** Moduli (G′, G′′) of SCM solution and SCM emulsion with different shear stresses. **f** Schematic illustration of the interfacial interaction in SCM emulsion. **g** Digital image of the SCM emulsion. Scale bar, 15 mm

Different from conventional nanomaterial stabilized emulsions, the fabrication of our robust SM_(O/W)_ hydrogels is free of ligands. It is reasonable to expect that the typical amphiphilic SF plays a vital role in the formation of stable Pickering emulsions by their specific adsorption characteristics [24]. In general, SF comprises 18 different amino acids, mainly including glycine (Gly), alanine (Ala), serine (Ser), and tyrosine (Tyr) [25, 26]. They repeatedly arrange to form two typical crystallizable (GAGAGS) and amorphous (GAGAGY) sequences, and their alternating arrangements yield heterogeneous electrostatic potential energies and amphiphilicity of the SF chains (Figs. 2a and S7). To validate the emulsification mechanism, we simulate the interactions between SF and toluene by using a molecular dynamics (MD) model (Fig. S8). Initially, the GAGAGS and GAGAGY sequences uniformly bond to the toluene molecules by hydrogen bonds and van der Waals, and only a slight deflection occurs within 100 ps remain, expecting the possibility of emulsion formation. To simulate the practical emulsion system, a composite model consisting of GAGAGS, GAGAGY sequences, toluene, and formic acid (numerical ratio, 2: 3: 500: 2,000) is proposed (Fig. 2b). The interactions of amino acids with different solvents are the basis of the emulsion systems in molecular dynamics. As expected, the hydrophilic amino acids (e.g., Ser and Tyr) of SF interact preferentially with FA molecules, whereas the hydrophobic amino acids and terminal groups (e.g., Gly, Ala, −CH_3_, and −C_6_H_6_) attract with toluene molecules (Fig. 2b2). The hydrogen bonds and van der Waals drive the GAGAGS and GAGAGY sequences to migrate from the edges toward the interior, where more solvent molecules are bound to stabilize the emulsion system (Fig. 2b1).

Furthermore, the binding energies of SF sequences with different solvents are quantificationally analyzed via DFT calculations (Fig. 2c, d). The hydrophilic amino acids make both GAGAGS and GAGAGY interact strongly with water molecules, as evidenced by their similar interaction energies (-123.38 *vs.* -121.42 kJ mol⁻^1^). Interestingly, the coexisting hydrophilic and hydrophobic groups deliver similarly strong interactions for SF sequences with FA (-61.43 and -58.83 kJ mol⁻^1^) and toluene molecules (-41.42 and -41.45 kJ mol⁻^1^). Therefore, the amphipathy of SF ensures the construction of FA-toluene interfaces in the metastable SF emulsion. Compared to the strong interfacial binding energy in traditional Pickering emulsions, the relatively weaker interfacial adsorption provided by amphiphilic SF chains fails to maintain stable in the long time. Evidently, the SF emulsion stratifies after 10 min (Fig. S9). Favorably, MXene nanosheets can help stabilize the two-phase interfaces, enabling the transition from a metastable SF emulsion to robust and sable sheet-stabilized Pickering emulsion. Otherwise, MXene dispersion without SF cannot form emulsions under shearing (Fig. S9). Consequently, the synergistic physical entanglement and barrier effects of SF chains and MXene nanosheets together reinforce and support the metastable interfaces of emulsion droplets (Figs. 2f and S10). Particularly, stiff MXene nanosheets conformably withstand the two-phase interfaces, and their polar surface groups (e.g., −O, −OH, and −F) can strongly interact with SF chains to stabilize the Pickering emulsion. In addition, the complementarity of the different dimensions allows for MXene and CNTs to improve the electrical conductivity of emulsion while avoiding early gelation, which easily occurs by using excessive MXene due to the too strong interactions with the SF chains. EDS confirms the uniform distribution of Ti_3_C_2_T_*x*_ MXene nanosheets on the skeleton of hydrogels (Fig. S11). As expected, the SF/CNTs/MXene (SCM) emulsion shows much larger liquid moduli than SCM solution owing to the reinforced interfacial interactions (Fig. 2e). The moduli (G′, G′′) (10^1^ ~ 10^2^ Pa) for the former is an order of magnitude higher than that of the later (10^0^ ~ 10^1^ Pa). Consistently, the SCM emulsion exhibits higher viscosity and more pronounced shear thinning behaviors (Fig. S12). Therefore, the flowable SCM emulsion precursors can flexibly produce the functional SCM_(O/W)_ hydrogels (Fig. 2g).

### Mechanical Properties and Structural Stability of Composite Hydrogels

**Fig. 3:**
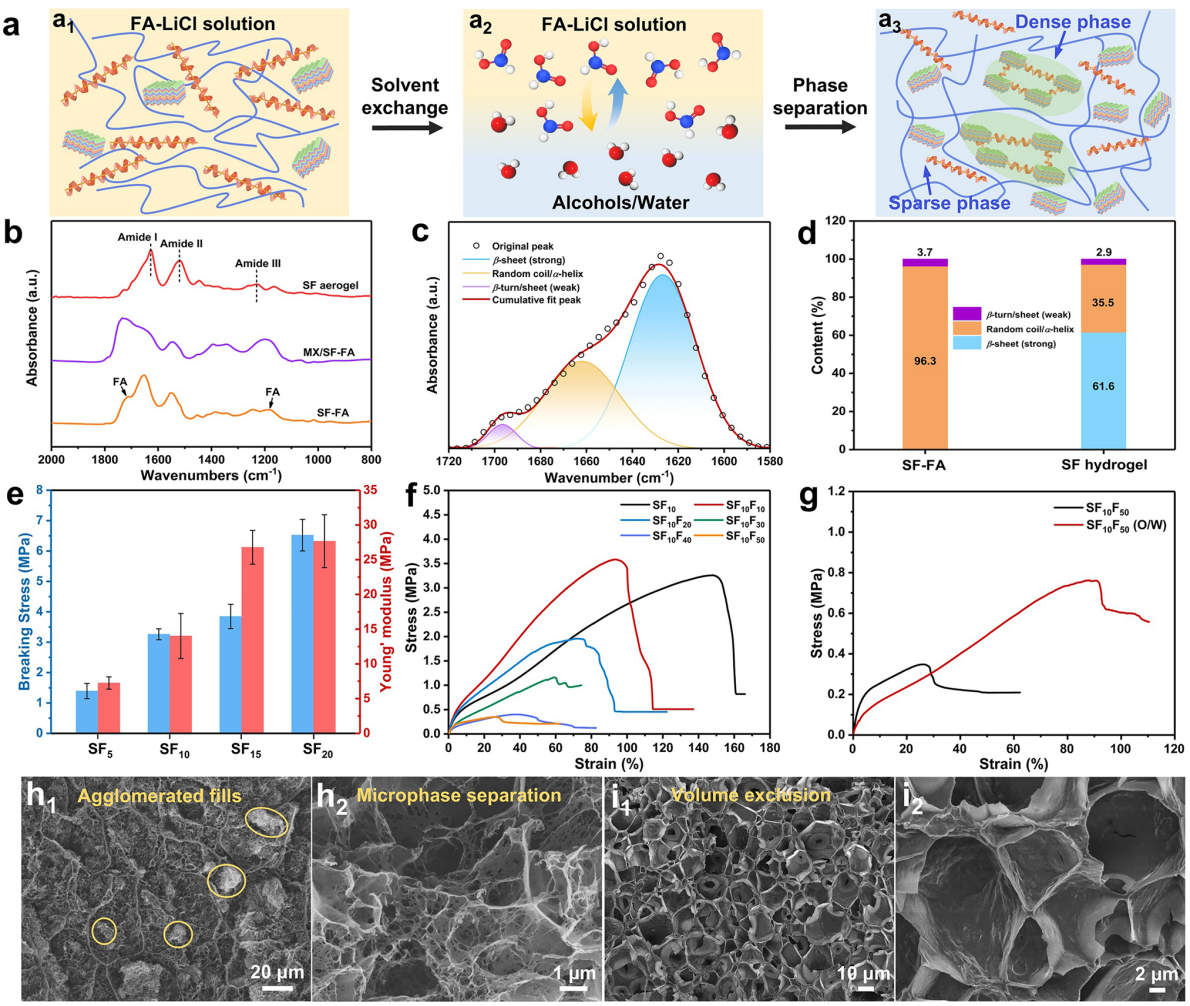
**a** Schematic illustrating the preparation process of silk fibroin hydrogel. **b** FTIR spectra of the SF-FA solution, MXene/SF-FA dispersion and SF hydrogel. **c** Deconvolution of the FTIR spectra in the amide I band of SF hydrogel. **d** Content of *β*-sheet, random coil/*α*-helix, and *β*-turn in SF-FA solution and SF hydrogel. **e** Strength and Young′ modulus of SF hydrogels. **f** Typical tensile stress–strain curves of SCM hydrogels with different filler contents. **g** Comparison of stress–strain curves for SF_10_F_50_ and SF_10_F_50_ (O/W) hydrogels. Cross sectional SEM images of **h** SF_10_F_30_ and **i** SF_10_F_30_ (O/W) hydrogels

To illustrate the formation mechanisms of SF hydrogel, we analyze the dynamic transformation of the secondary amorphous (*α*-helix and random coil) and crystalline (*β*-sheet crystal) structures of SF during the solvent exchange process [27]. Initially, the LiCl-FA solution swells the SF fibril and destroys its *β*-sheet crystalline to form homogeneous SF solution, where the intermolecular hydrogen bonds are weakened by the hydration effect of strong polar ions (Li^+^) (Fig. 3a1). During the solvent exchange process, the added poor solvents (e.g., water, alcohols, and other polar solvents) reconstruct the intermolecular hydrogen bonds of the SF chains (Fig. 3a2). Concomitantly, the microphase separation of SF chains induces the separation of dense and sparse phases in the SF hydrogel (Fig. 3a3). At the same time, the secondary conformation transits from random coil to stable *β*-sheets, and the adjacent *β*-sheets gradually assemble into *β*-crystalline (Fig. S13). This enables the self-assembly and gelation of SF chains, and confers high tensile strength and structural stability to the hydrogel. In other words, the *α*-helix and random coil, and *β*-sheet nanocrystal constitute the sparse and dense regions of the SF hydrogel, respectively. Consequently, the solvent exchange induces microphase separation and self-cross-linking of SF chains to form SF hydrogels owing to the affinity variation (Fig. 3a). Accordingly, the SCM emulsions are converted into hydrogels by a simple solvent exchange. The conformational transition and gelation processes of the hydrogels are monitored by FTIR spectra (Fig. 3b). Clearly, the presence of silk fibroin is validated by the typical amide I (1700 − 1600 cm^–1^), amide II (1600 − 1500 cm⁻^1^), and amide III (1300 − 1200 cm⁻^1^) bands. By comparison, the disappearance of the FA peaks in the SF hydrogel verifies the successful solvent exchange. By deconvolving the amide I band, the secondary conformational changes before and after gelation are quantified (Figs. 3c and S15). Initially, SF-FA solution contains predominate amorphous structure (96.3%) and only few *β*-turn/sheet structure (3.7%). After solvent exchange, the amorphous structure accounts for 35.5%, but *β*-sheet crystal structure for 61.6% with few *β*-turn/sheet (2.9%) in the obtained hydrogel (Fig. 3d).

These favorable secondary conformations confer SF hydrogels with outstanding mechanical properties. In specific, the amorphous structures afford elasticity and toughness, while the crystalline structures mainly provide strength and hardness. Notably, more SF chains can help enhance mechanical properties of SF hydrogels by amplifying the intermolecular interactions and secondary structures (Figs. 3e and S16). For instance, the tensile strength and Young’ modulus are ~ 1.4 MPa and ~ 1.3 MPa at 5% SF, which increases to ~ 6.5 MPa and ~ 27.7 MPa for 20% SF, respectively. The cross sectional SEM image of fractured SF hydrogel reveals the internal dense network and oriented structure (Fig. S17a). For the composite hydrogels, their mechanical properties are explored in terms of nanofiller (MXene and CNTs) content (F*x* wt%) in 10% SF matrix (Fig. 3f). Reasonably, low contents of nanofillers improve the breaking stress from ~ 3.3 to ~ 3.6 MPa (Fig. S17b), but the dramatic deterioration of the mechanical properties and shape stability are caused by the formed agglomerates and fracture sites at high filler loadings (Figs. 3g and S17c, d). Interestingly, the emulsion templates can optimize the internal structure by constructing stable two-phase Pickering interfaces and avoid obvious random filler agglomeration, particularly at high filler levels (Fig. 3h). Consequently, the reinforced interfaces and ordered pores in O/W hydrogels effectively transfer internal stresses and support the macroscopic framework (Figs. 3i and S18), affording remarkable bendability and flexibility for hydrogels (Fig. S19).

### Electrical and EMI Shielding Performance of Composite Hydrogels

**Fig. 4:**
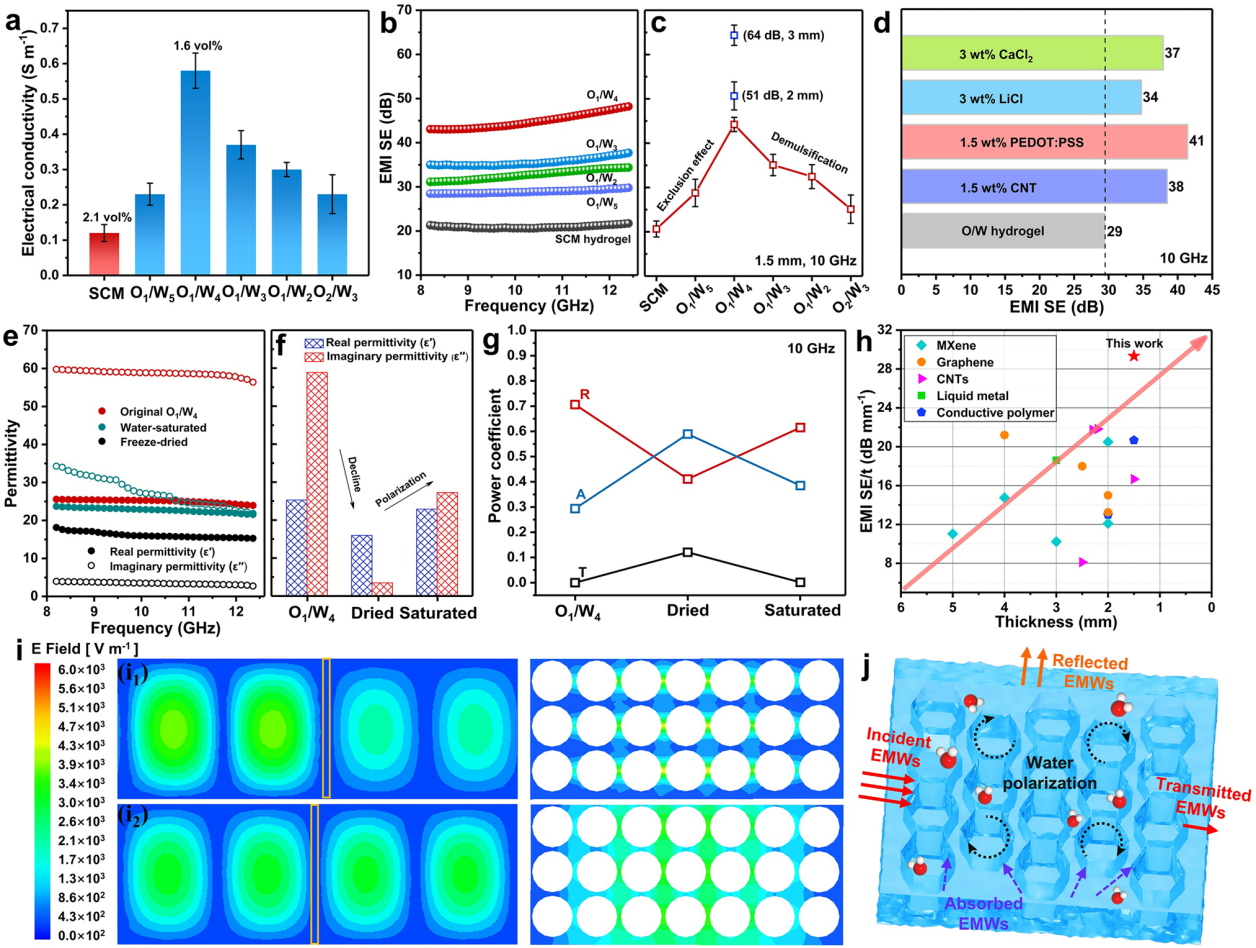
**a** Electrical conductivity and **b, c** EMI shielding properties of SCM and SCM_(O/W)_ hydrogels with different toluene volume fractions. **d** Comparison of EMI SE values at 10 GHz for SCM_(O/W)_ hydrogels assembled by different conductive components. **e, f** Dielectric parameters (real and imaginary permittivity) and **g** power coefficients of SCM_(O1/W4)_ samples in different states. **h** Comparison of the EMI shielding performance of SCM_(O/W)_ hydrogel with other polymer-based composites. **i** Simulated energy distribution of electric field for SCM_(O1/W4)_ **i**_**1**_ hydrogel and **i**_**2**_ aerogel. **j** Schematic illustrating for EMI shielding mechanism. In general, the SF content is 5% and the thickness of all studied objects is 1.5 mm

Except for the intriguing mechanical performance, the optimized conductive structures, together with water polarization effect, grant attractive EMI shielding properties to the SCM_(O/W)_ hydrogels. By controlling the volume fraction of oil phase (~ 0.17, 0.2, 0.25, ~ 0.33, 0.4), the obtained SCM_(O/W)_ hydrogels are denoted as O_*x*_/W_*y*_, where *x* and *y* represent volume ratios of two phases. Interestingly, the SCM_(O/W)_ hydrogels show much higher electrical conductivity than SCM hydrogel (~ 0.12 S m^–1^, 2.1 vol%) owing to the optimized conductive network (Fig. 4a). As the oil phase content increases, the conductivity of SCM_(O/W)_ hydrogel gradually improves and an optimum value is obtained at the *φ* = 0.2 (~ 0.58 S m⁻^1^, 1.6 vol%). However, further increment in volume content reduces the electrical conductivity owing to the continuous structure disruption. In accordance, EMI shielding performance of SCM_(O/W)_ hydrogels also follow the similar variation trend (Fig. 4b). Specifically, the volume exclusion effect improves EMI shielding effectiveness (SE) from ~ 21 to ~ 44 dB as *φ* increases from 0 to 0.2 (Fig. 4c). Reasonably, appropriate volume contents of oil phase help form dense conductive network (Figs. S4 and 5a), but excess oil contents trigger demulsification. The porous structure is partially transformed from close- to open-celled morphology (Fig. S20), deteriorating the continuous conductive network in the hydrogels. The size effect (thickness) results in a significant increase in the EMI shielding performance of hydrogels, as evidenced by the EMI SE values of ~ 51 and ~ 64 dB at 2 and 3 mm, respectively (Fig. 4c). Inspiringly, the inclusion of functional nanoparticles and free ions, e.g., CNT, PEDOT: PSS, LiCl, and CaCl_2_ solution, in the hydrogels during solvent exchange endows required functionalities, such as electrical conductivity and EMI shielding capability (Figs. 4d and S21). For example, the EDS elemental mapping images reveal the uniform distribution of Ca^2+^ ions inside hydrogel with CaCl_2_ solution for solvent exchange (Fig. S11).

To clarify underlying shielding mechanism of hydrogels, we explore the influence of water phase on EMI shielding properties of the SCM_(O/W)_ hydrogels. Clearly, the freeze-dried SCM and SCM_(O1/W4)_ aerogels (F-aerogel) show reduced EMI SE values of ~ 7 and ~ 15 dB as compared to their hydrogel counterparts (~ 21 and ~ 44 dB) (Fig. S22). After dehydration, the shielding performance of samples mainly derives from the conductive framework, and the optimized structure (O/W aerogel) exerts stronger attenuation ability of EMWs. Subsequently, the backfill of water in the aerogels reconverts them into the hydrogel counterparts (W-hydrogel), to some extent, which reversely improves the EMI SE to ~ 22 dB for W-SCM hydrogel and ~ 31 dB for W-SCM_(O1/W4)_ hydrogel owing to the synergistic effect of the conductive network and water polarization (Fig. S22). Of course, some irreversible structure destruction during the above processes causes partial performance degradation, making the W-hydrogels show slightly inferior shielding properties as compared to their precursor hydrogels.

The power coefficients (reflection, absorption, and transmittance coefficients, R-A-T) and complex permittivity are tested to analyze the shielding mechanisms [28, 29]. The real and imaginary permittivity (ε′ and ε′′) characterize the polarization and loss capabilities of materials, respectively [30, 31]. As expected, the desorption and absorption of water in the composite hydrogels dynamically modulate the polarization and loss characteristics (Fig. 4e, f). Typically, the ε′ (10 GHz) initially decreases from ~ 25 to ~ 16 upon lyophilization, and then regrows to ~ 23 after water saturation. Accordingly, the ε′′ plunges from ~ 59 to ~ 3 and then reverts to ~ 27 in the same processes (Fig. 4f). Interestingly, the ε′′ value is larger than the ε′ value either for SCM_(O1/W4)_ or W-hydrogel, giving higher polarization loss (tanδ = ε′′/ε′). It is reasonably understood that water phase can effectively connect conductive pathways and provide strong interfacial polarization to enhance the loss capability against EMWs for the hydrogels. In contrast, the ε′′ value is smaller than the ε′ value for the F-aerogel upon dehydration treatment, which drastically weakens the EMI shielding ability. In accordance, the variations of power coefficients and absorption (absorption loss/reflection loss, SE_A_/SE_R_) consist with the above results (Figs. 4g and S23). Consequently, these results highlight the significant contribution of water to the excellent EMI shielding properties in the SCM_(O1/W4)_ hydrogels, which synergistically strengthens interfacial polarization and optimizes conductive structure (Fig. 4j). More importantly, our emulsion-based strategy is advantageous in tuning microstructures and properties of the hydrogels as compared to conventional techniques, which rely much on high loadings of fillers. As shown in Fig. 4h and Table S1, the composite SCM_(O/W)_ hydrogels demonstrate both superior EMI SE and thin thickness comparable to other state-of-the-art polymer-based EMI shielding materials [32–35].

According to Maxwell’s equations, the incident EMWs induce charge oscillation on conductive material surface, which in turn interact with and attenuate the EMWs. To validate the shielding characteristics, the electric field distributions of EMWs in the waveguide cavity are simulated by finite element analysis (Fig. 4i). Compared to the F-aerogel, the SCM_(O/W)_ hydrogel more effectively inhibits the transmission of EMWs, leading to a reduced electric field distribution on the surface. In contrast, the electric field intensity remains almost constant for the aerogel. Besides, the electric field distribution undergoes a significant reduction at the middle position in deionized water medium, which further demonstrates the strong attenuation of EMWs by water polarization (Fig. S24).

### Solar-Driven Evaporation Performance of Composite Hydrogels

Except for the attractive EMI shielding performance, the solar-driven water evaporation properties can also be promoted through the precise regulation of hierarchical structures of the hydrogels. It allows a rational trade-off between the efficiency of mass and heat transfer and light-to-heat energy efficiency in hydrogel. Usually, traditional hydrogel evaporators are freeze-dried to construct homogeneous small pore structure (< 10 μm) or vertical channels inside them to enhance water evaporation performance [36–39]. However, emulsion template and microphase separation not only construct large open-celled skeleton (10–100 μm) for SCM_(O/W)_ hydrogel, but also preserve the small pore structure (~ 1 μm) in the cell walls (Fig. S20). On the one hand, the small pores with strong capillary force facilitate the internal water transport of SCM_(O/W)_ hydrogel evaporators. On the other hand, the large pores greatly reduce the water content of hydrogel to prevent significant heat loss from heating water phase and attenuate downward heat transfer to maintain an efficient evaporating surface. Meanwhile, the open-celled structures of SCM_(O/W)_ hydrogels endow the rapid vapor escape and efficient heat homogenization within hydrogels.

**Fig. 5:**
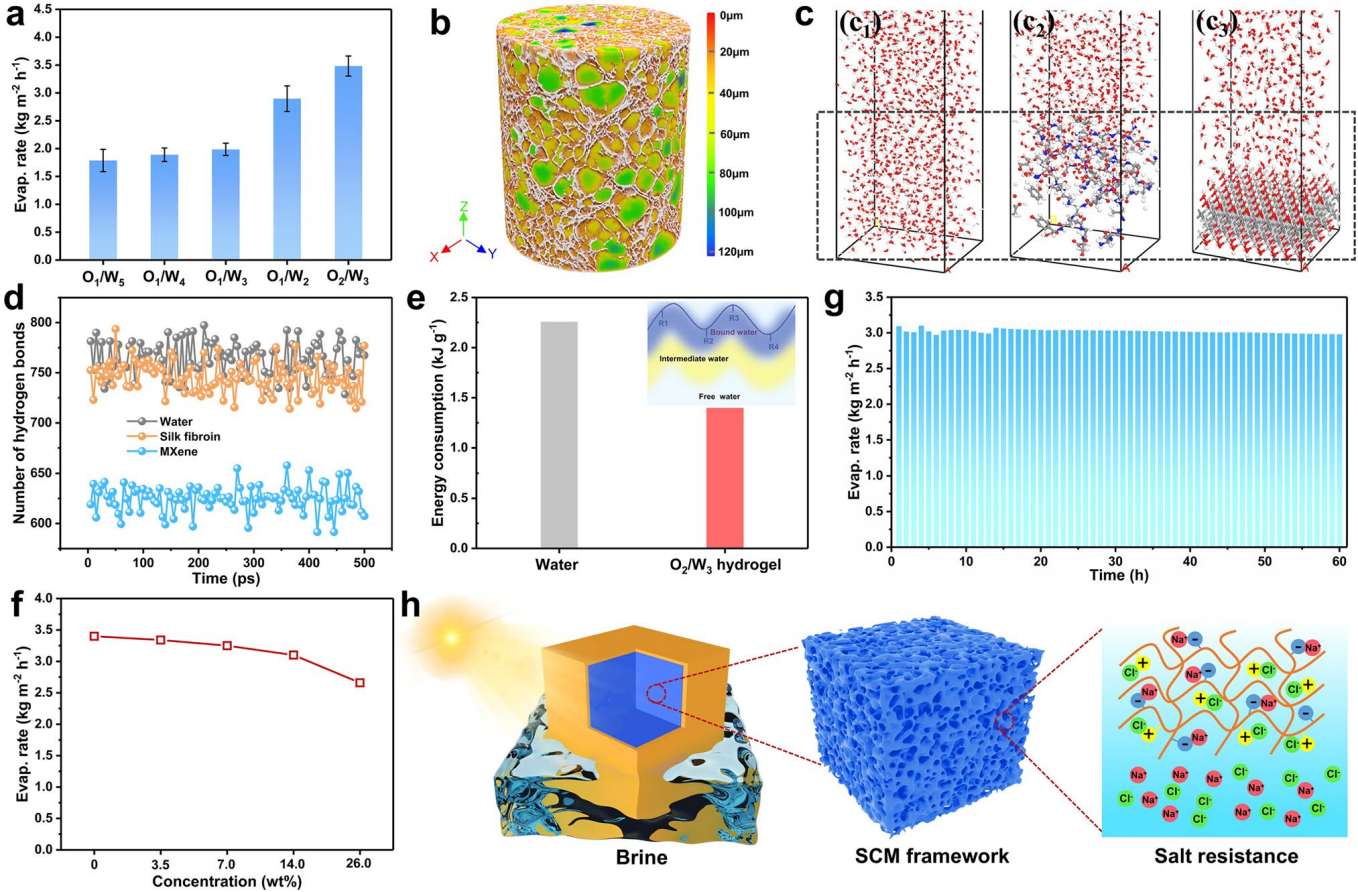
**a** Water evaporation rates for hydrogel generators with different toluene volume ratios. **b** 3D reconstruction of the porous aerogel via micro-CT technique, different colors represent different pore sizes. **c** MD simulations of **c**_**1**_ water–water, **c**_**2**_ silk fibroin–water, and **c**_**3**_ MXene-water systems at 500 ps snapshots. **d** Number of hydrogen bonds between water molecules within 500 ps of different systems. **e** Energy consumption of water and SCM_(O2/W3)_ hydrogel generator. **f** Evaporation rates of samples in different brines. **g** Evaporation rate of SCM_(O2/W3)_ hydrogel in 14 wt% brine during the cycling experiment. **h** Salt resistance mechanism of hydrogel evaporator

The intriguing solar-thermal responses of MXene and CNTs lend strong light-absorbing capacity to the hydrogel evaporators. As a result, the SCM_(O2/W3)_ hydrogel generator shows a high surface temperature of 38.6 ℃ under 1-sun simulated irradiation at wet conditions (Fig. S25). The mass change and water evaporation rate curves of hydrogel evaporators are determined in a specific test system (Fig. S26). The water evaporation rate is calculated from the slope of mass change curve (Fig. S27a, b). The evaporation rate of hydrogel improves with the increment in volume fraction of oil phase, the SCM_(O2/W3)_ hydrogel gives the highest value of ~ 3.5 kg m^−2^ h^−1^ owing to the optimized water transport channels and economical energy consumption (Fig. 5a). To uncover the underlying mechanisms, SEM and micro-CT analysis visualize the porous structures of SCM_(O2/W3)_ hydrogel (Figs. 5b and S20). Different from the clos-celled structure of SCM_(O1/W4)_ hydrogel, SCM_(O2/W3)_ hydrogel shows partially open porous structure when the volume fraction of oil phase beyond the emulsion threshold. The open penetrable channels allow the solar-thermal derived water vapor to freely escape and evaporate out of the hydrogel. Besides, the faster water evaporation of SCM_(O2/W3)_ hydrogel compared to the SCM hydrogel (~ 3.5 *vs.* ~ 1.4 kg m⁻^2^ h⁻^1^) highlights the significance of emulsion-templated porous structure (Fig. S27c, d). Also, the water evaporation properties can be further optimized and flexibly regulated by Pickering emulsion template.

Moreover, the evaporation enthalpy significantly influences the water evaporation performance. Generally, the physical states of water in the hydrogel evaporators mainly include bound water, intermediate water, and free water [40]. The bound water is tightly bound to the hydrophilic cross-linked polymer network by hydrogen bonds, and the intermediate water bound by fewer hydrogen bonds easily evaporates during the solar steam generation. Thus, the decreased hydrogen bonds reduce the evaporation enthalpy and accelerates the water evaporation of evaporators. We simulate the hydrogen bonds of SCM_(O2/W3)_ hydrogel by MD simulation (Fig. 5c, d). The amino acids of SF and the surface polar terminations of MXene help trap water molecules by hydrogen bonds or electrostatic forces. Significantly, it is the hydrogen bonding and intermolecular forces that can ultimately modulate the phase transition behaviors of water and afford efficient water evaporation [4]. Accordingly, SCM_(O2/W3)_ hydrogel evaporators consume much lower energy than the direct evaporation of bulk water (~ 1.4 *vs.* ~ 2.3 kJ g^–1^), enabling much faster water evaporation with an evaporation efficiency of 92.2% (Fig. 5e). Unlike traditional fabrication methods of hydrogels, our Pickering emulsion strategy can flexibly and precisely tune the cellular morphology of hydrogels and ultimately the evaporation performance. The continuous skeleton ensures rapid water uptake, and the open-celled structure provides the hydrogel with excellent heat consumption management by vapor escape. Hence, the prepared hydrogel manifests obvious advantages over the reported hydrogel evaporators (Table S2, Fig. S28) [41–43].

In practical applications, the capacity for high salt tolerance is an essential characteristic for water evaporators to handle highly concentrated brines. It is crucial to guarantee the efficient and reliable operation of evaporators under conditions of elevated salinity. So, we explore the evaporation rates of SCM_(O2/W3)_ hydrogels in different simulated brines (NaCl solution). Although the evaporation rate of brine in the hydrogel decreases as the concentration of NaCl increases, it still retains a competitive evaporation rate of over 2.5 kg m⁻^2^ h⁻^1^ when using a 26 wt% NaCl solution (Fig. 5f). Typically, SCM_(O2/W3)_ hydrogel keeps a relatively stable evaporation rate in 14 wt% simulated brine for 60 h (Fig. 5g). The salt crystallization occurs mainly around the sample, which would not block the light and water transport channels, and therefore compromise its evaporation performance (Fig. S29). Two underlying mechanisms for the salt tolerance of the hydrogel are proposed (Fig. 5h). Firstly, the amphiphilic SF chains are capable of simultaneously interacting with both Cl⁻ and Na⁺ ions, leading to a strengthened anti-polyelectrolyte effect in brine, thereby inhibiting the salt crystallization process. Secondly, the hierarchical transport channels, characterized by the coexistence of both large and small pores, facilitate the horizontal diffusion of salt components in response to the concentration gradient.

## Conclusions

In summary, we describe a general strategy to construct customizable composite hydrogels and confer them with the optimized micro-structure and functionalities based on Pickering emulsion and microphase separation, which greatly alleviate the common yet significant agglomeration in polymers induced by increasing nanofillers. Also, the robust two-phase interfaces by means of synergistic effect of amphiphilic polymers and MXene nanosheets are prepared without any surfactant. The variable oil phase content determines the closed and open pore structures of SCM_(O/W)_ hydrogels, and effectively regulates their macroscopic performance. The ordered conductive network and the strong water polarization allow SCM_(O/W)_ hydrogels with excellent EMI shielding properties. The interconnected open-celled structures of SCM_(O/W)_ hydrogels endow the rapid vapor escape and efficient heat homogenization within hydrogels. Also, the hierarchical transport channels and the anti-polyelectrolyte effect induced by the amphiphilic SF realize the satisfactory brine evaporation rate of composite hydrogels.

## Supplementary Information

Below is the link to the electronic supplementary material.

Supplementary file1 (DOC 38788 KB)
