# Supplementary material for: Pickering Emulsion-Driven MXene/Silk Fibroin Hydrogels with Programmable Functional Networks for EMI Shielding and Solar Evaporation
Source: Nanomicro Lett. 2025 Jun 24;17:312. doi: 10.1007/s40820-025-01818-w (PMC12185816; doi:10.1007/s40820-025-01818-w)
Supplement: Supplementary file 1 — Supplementary file1 (DOC 38788 KB) [file 40820_2025_1818_MOESM1_ESM.doc]

Supporting Information for

**Pickering Emulsion-Driven MXene/Silk Fibroin Hydrogels with Programmable Functional Networks for EMI Shielding and Solar Evaporation**

Guang Yin^1, 2, #^, Jing Wu^1, #^, Chengzhang Qi^2^, Xinfeng Zhou^2^, Zhong-Zhen Yu^1, 2^, Hao-Bin Zhang^1, 2^, *

^1^ State Key Laboratory of Organic-Inorganic Composites, Beijing University of Chemical Technology, Beijing 100029, P. R. China

^2^ Center for Nanomaterials and Nanocomposites, College of Materials Science and Engineering, Beijing University of Chemical Technology, Beijing 100029, P. R. China

^#^Guang Yin and Jing Wu have contributed equally to this work.

*Corresponding author. E-mail: [zhanghaobin@buct.edu.cn](mailto:zhanghaobin@buct.edu.cn) (Hao-Bin Zhang)

**Supplementary Figures and Tables**


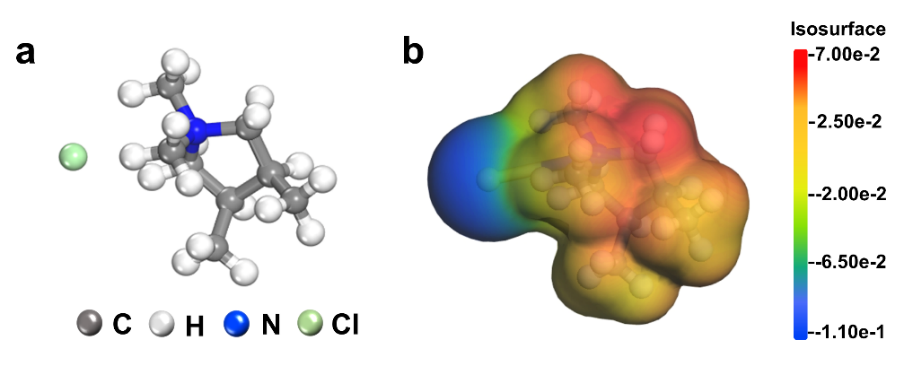


**Fig. S1** (**a**) The PDDA molecule and (**b**) its electrostatic potential energy


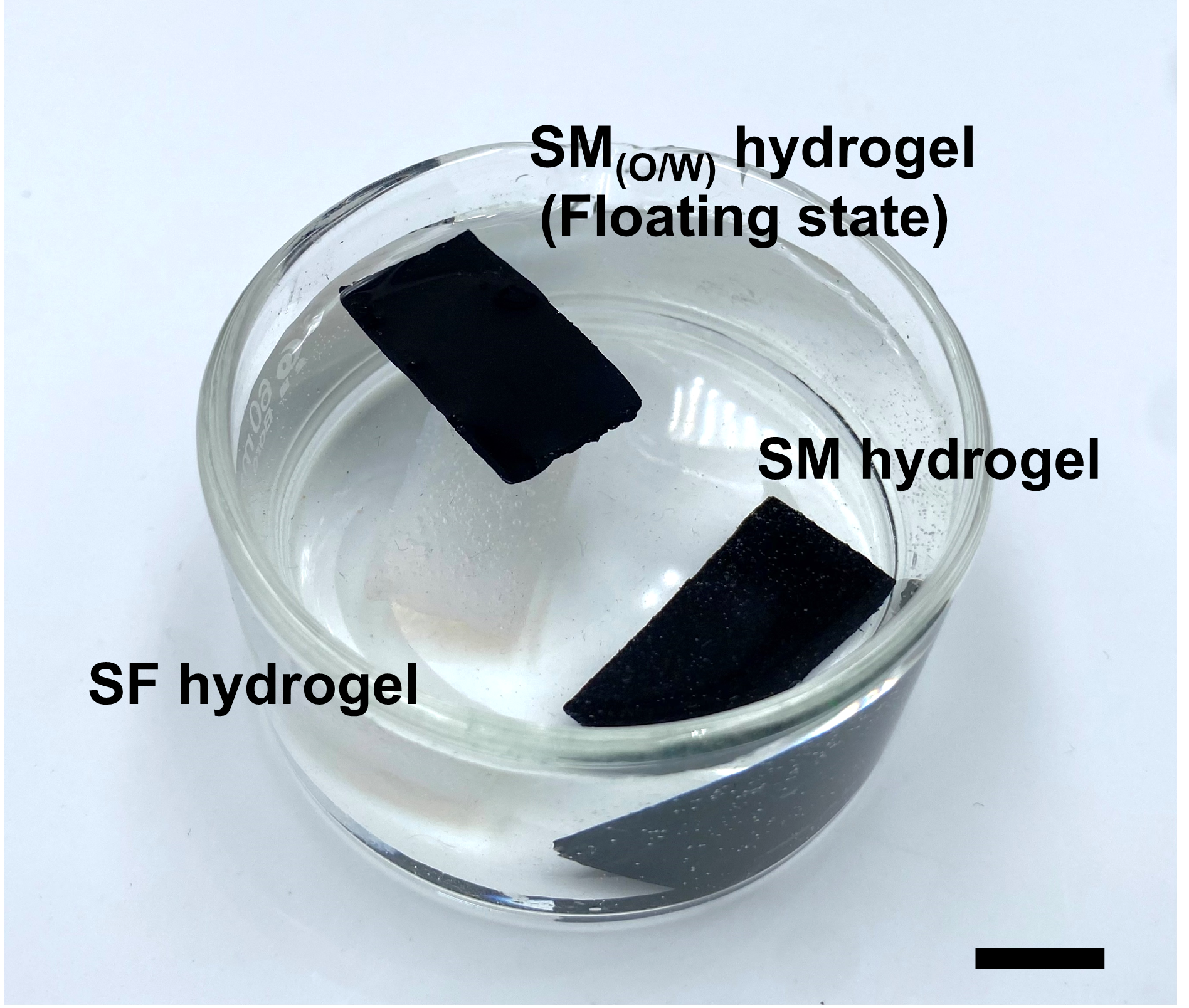


**Fig. S2** The digital photographs of SF, SM and SM_(O/W)_ hydrogels. Scale bar, 15 mm


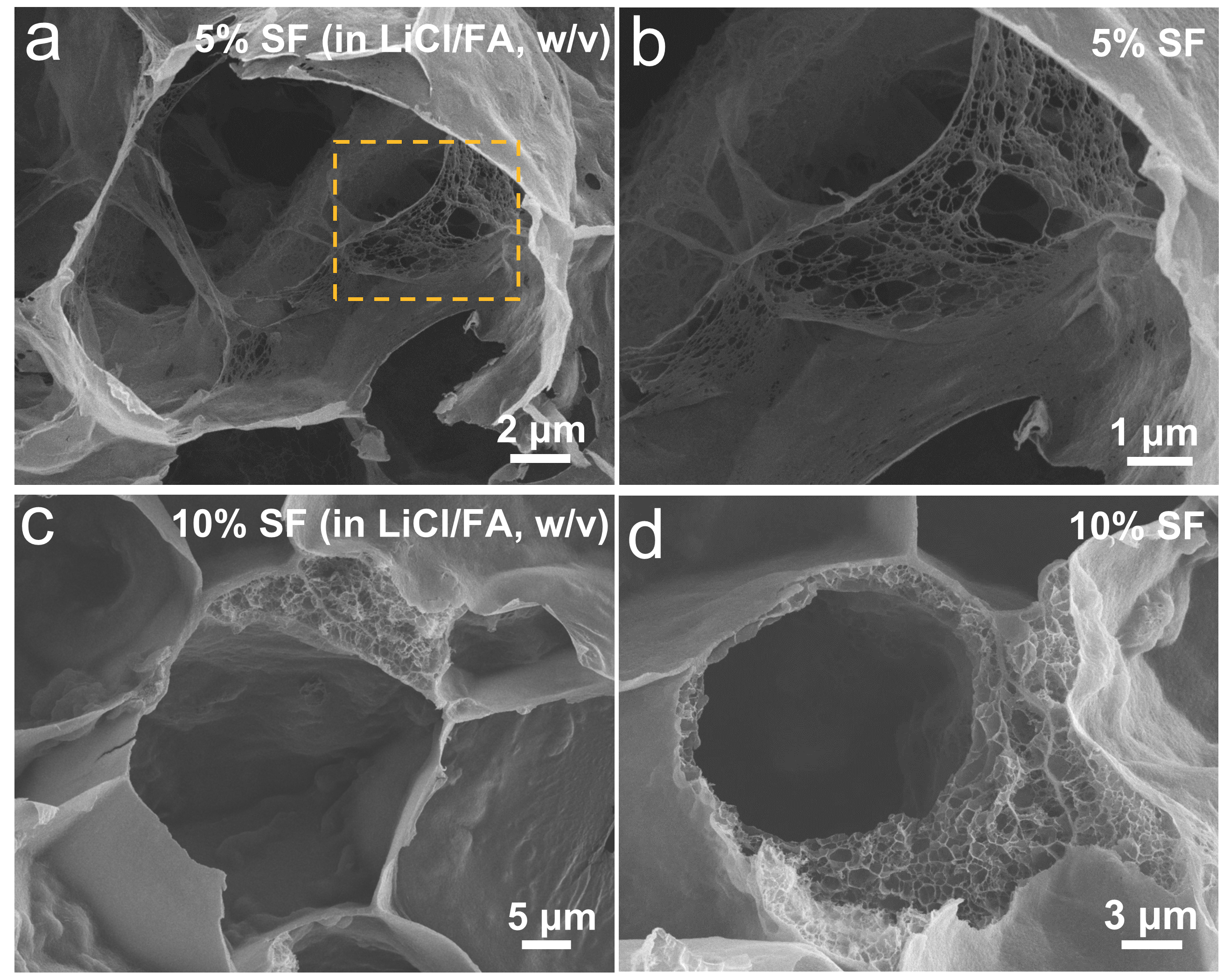


**Fig. S3** Phase separation structures of SM_(O/W)_ hydrogels with SF content of (**a-b**) 5% and (**c-d**) 10%, respectively


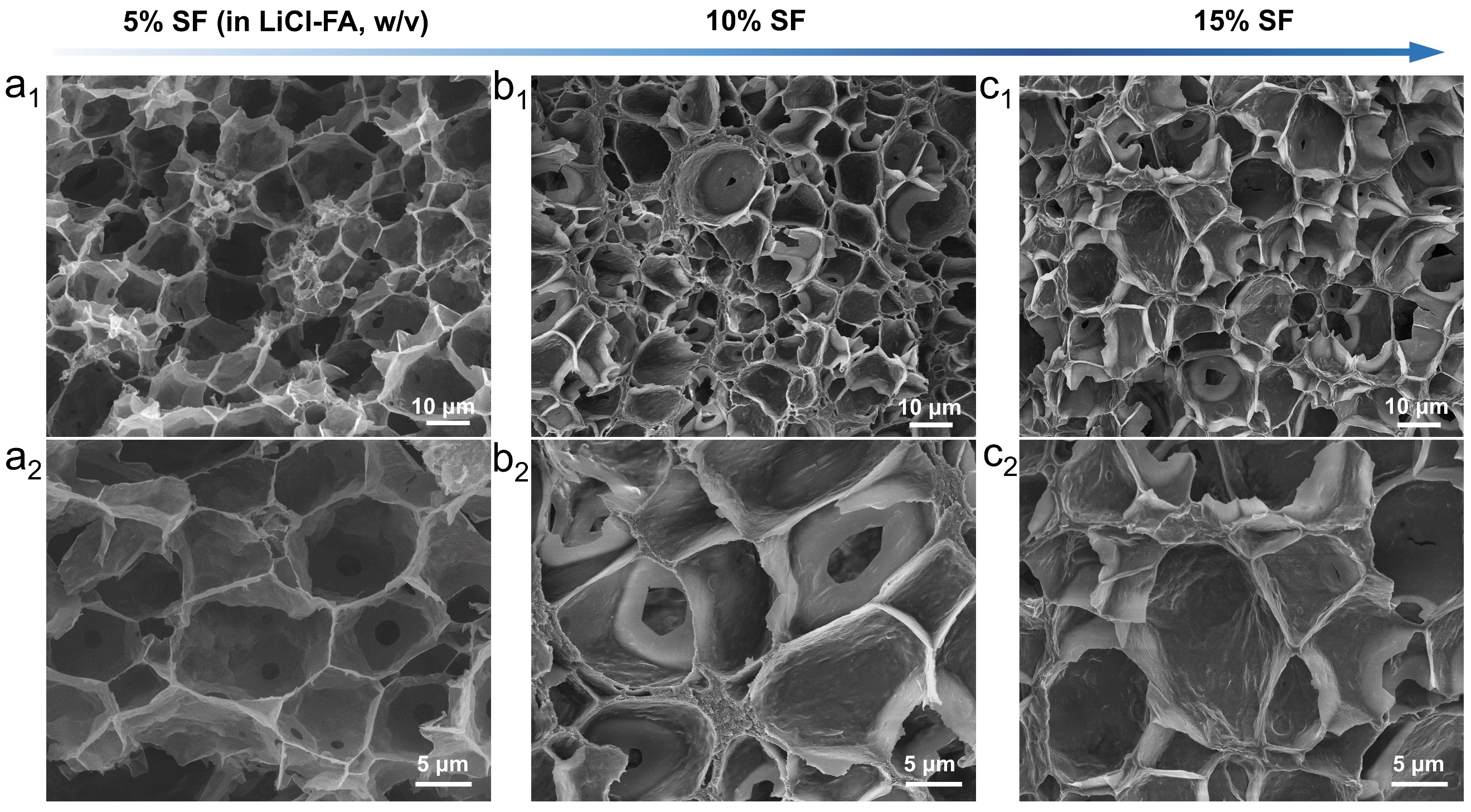


**Fig. S4** SEM images of SM_(O/W)_ hydrogels with SF content of (**a**) 5%, (**b**) 10%, and (**c**) 15%, respectively


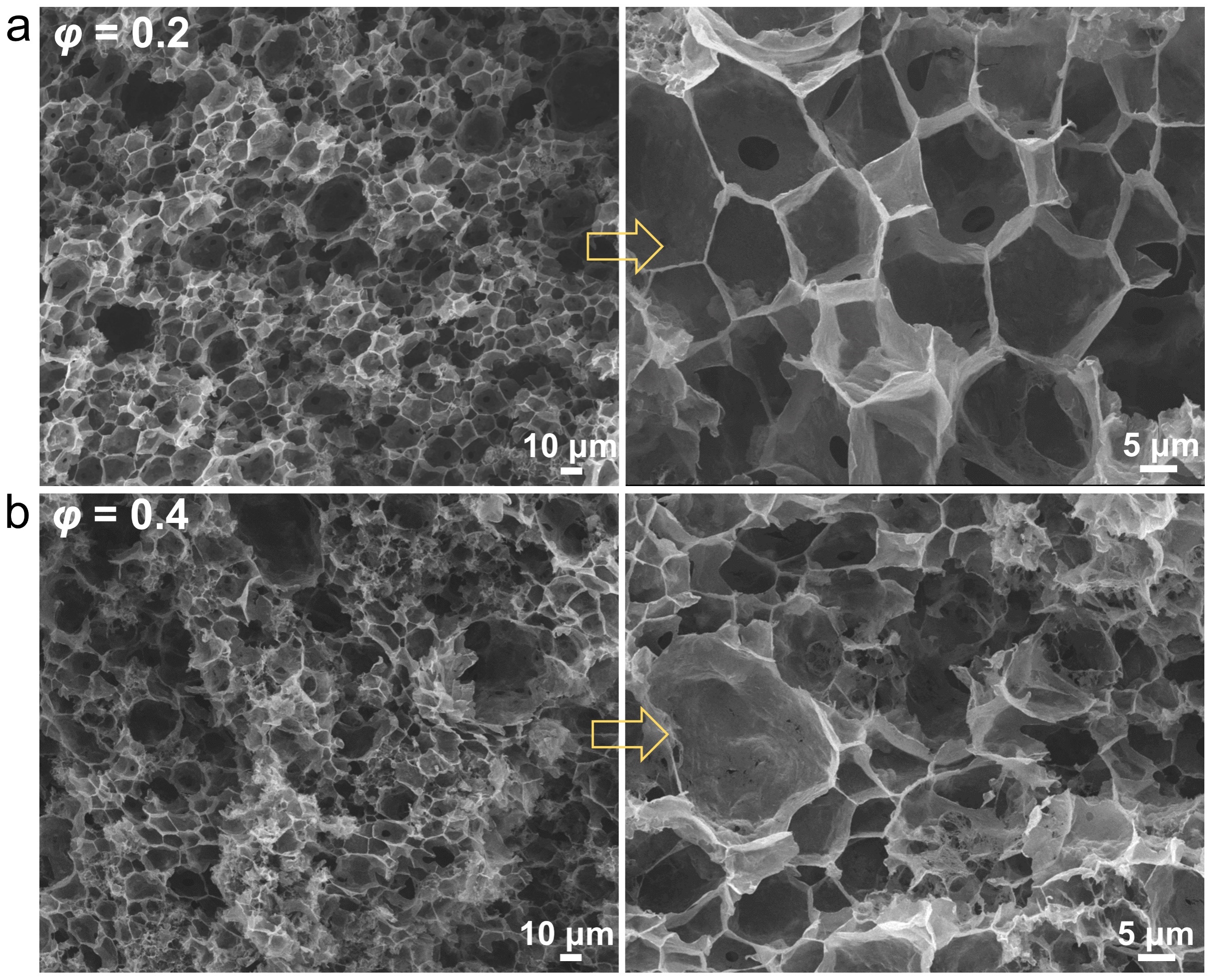


**Fig. S5** SEM images of SM_(O/W)_ hydrogels with oil phase volume fraction of (a) 20% and (b) 40%


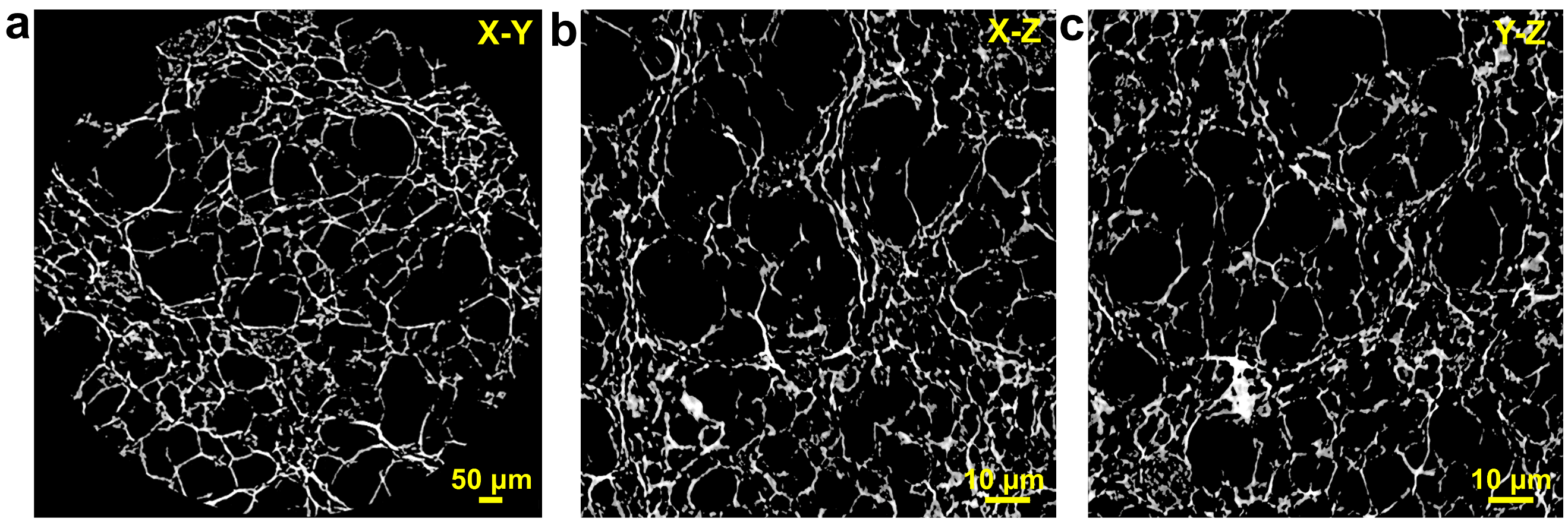


**Fig. S6** 2D cross-section images of SM_(O/W)_ hydrogels in (a) X-Y, (b) X-Z, and (c) Y-Z directions


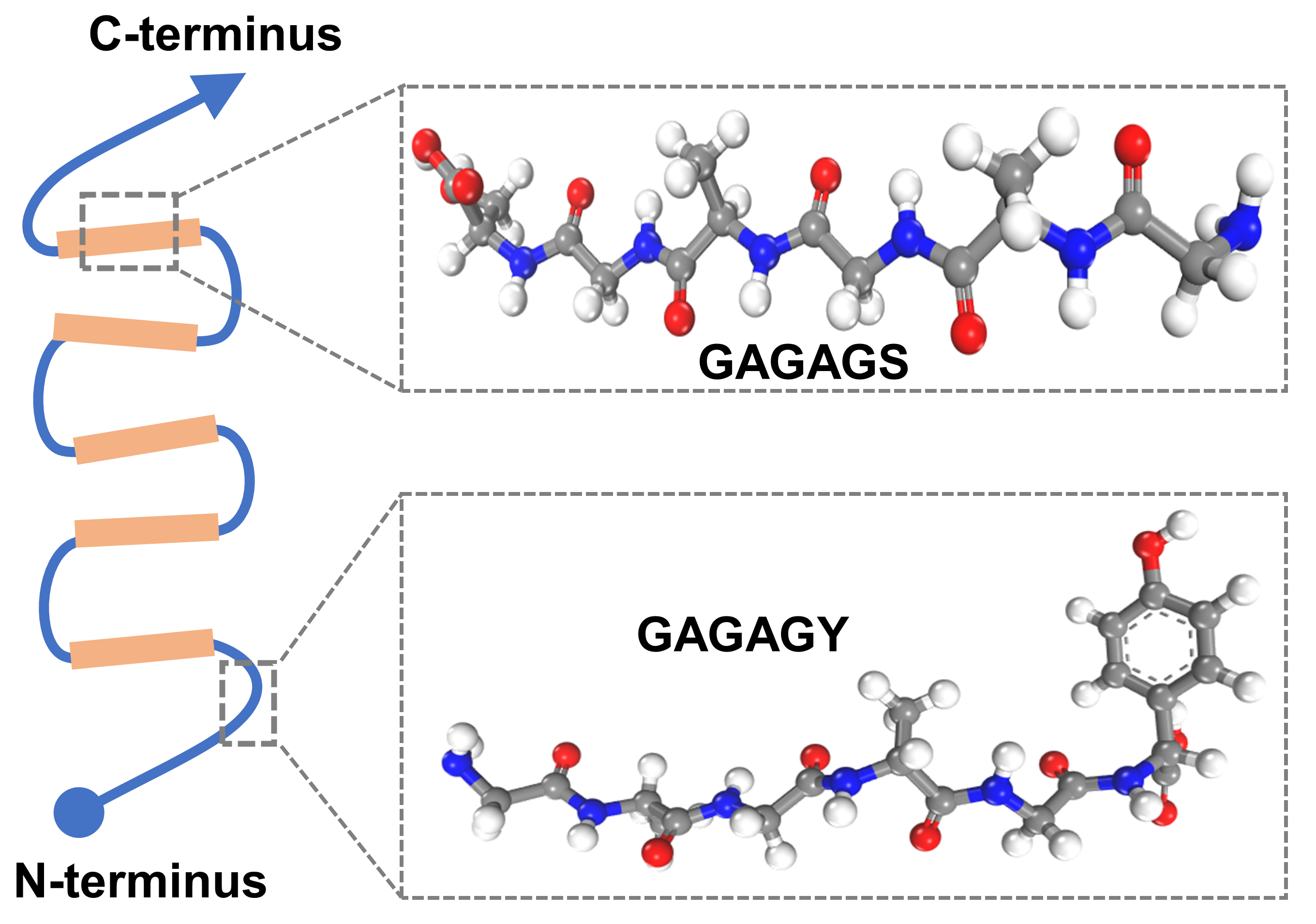


**Fig. S7** The crystallizable sequence (GAGAGS) and amorphous sequence (GAGAGY) in heavy-chain (H-chain)


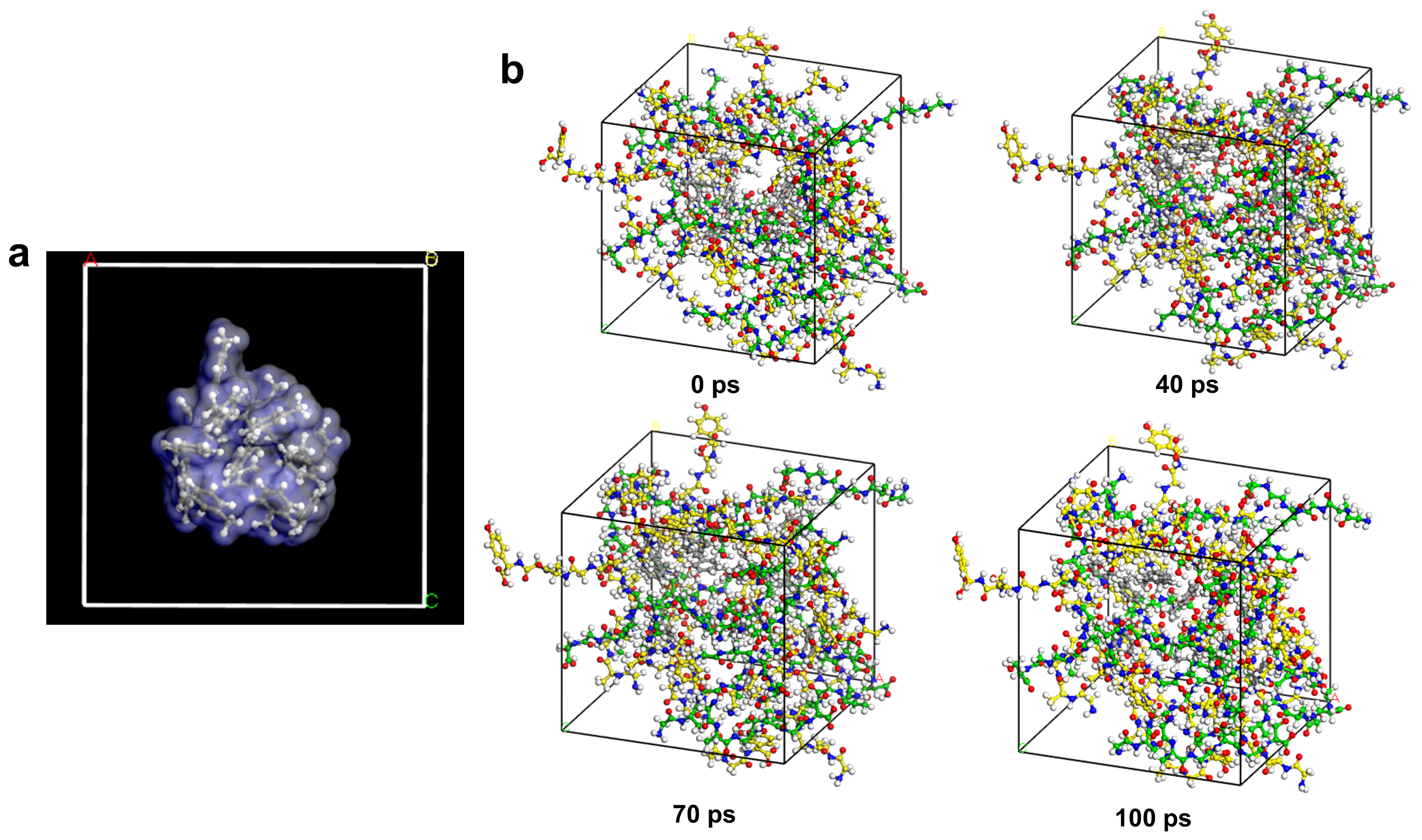


**Fig. S8** (**a**) The individual toluene cluster model. Molecular dynamics simulation of silk fibroin-toluene unit at (**b**) 0, 40, 70, and 100 ps snapshots


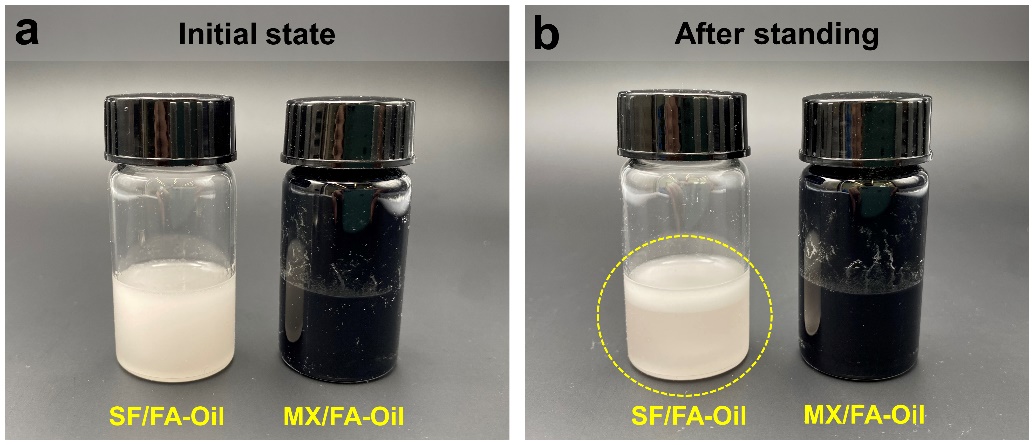


**Fig. S9** The digital photographs of SF/FA-toluene and MXene/FA-toluene mixture in the (**a**) initial and (**b**) after-standing states. (The MXene/FA-toluene without SF cannot form emulsion under shearing.)


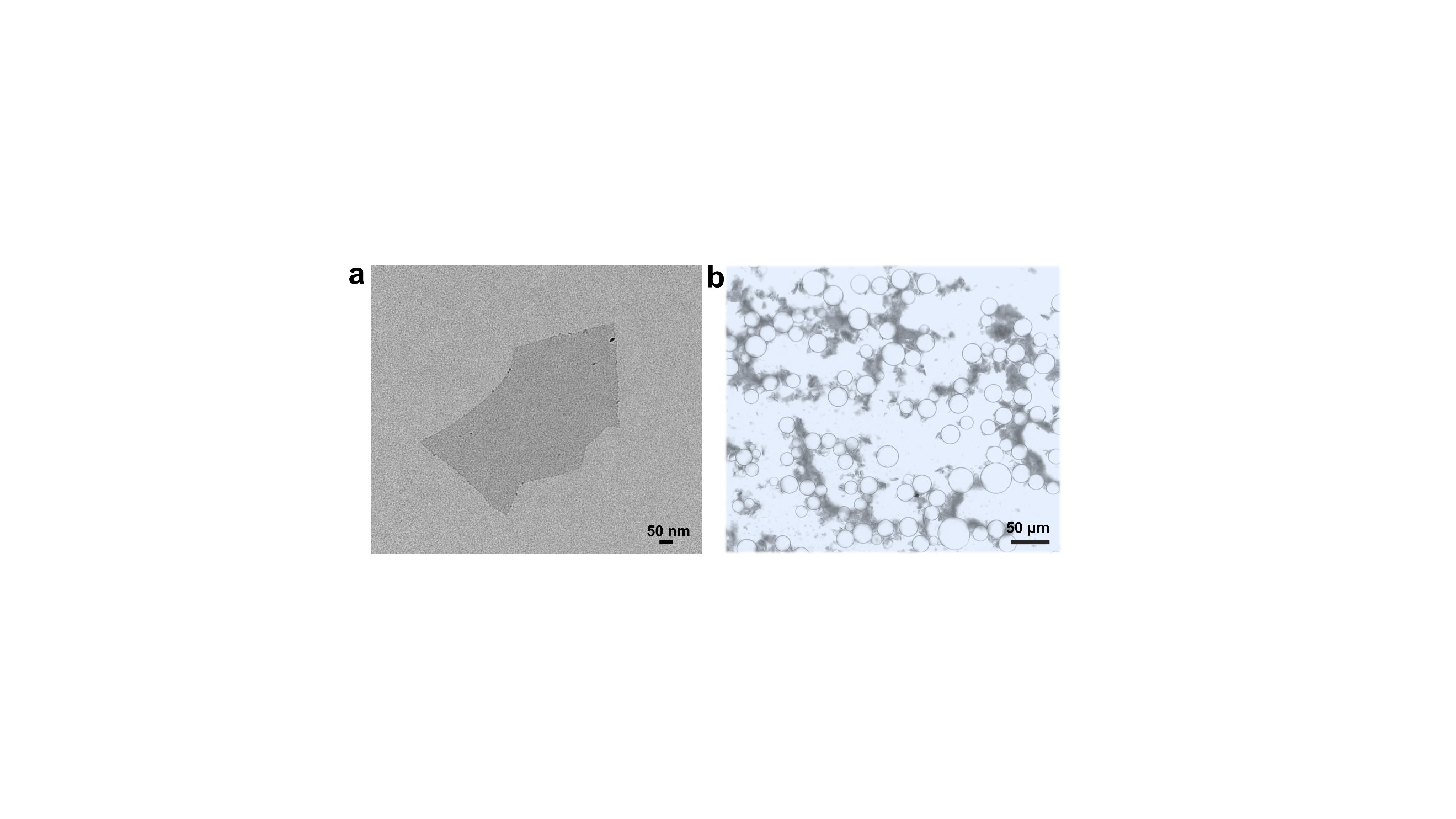


**Fig. S10** (**a**) TEM image of MXene nanosheets and (**b**) the optical microscope image of SF/MXene emulsion


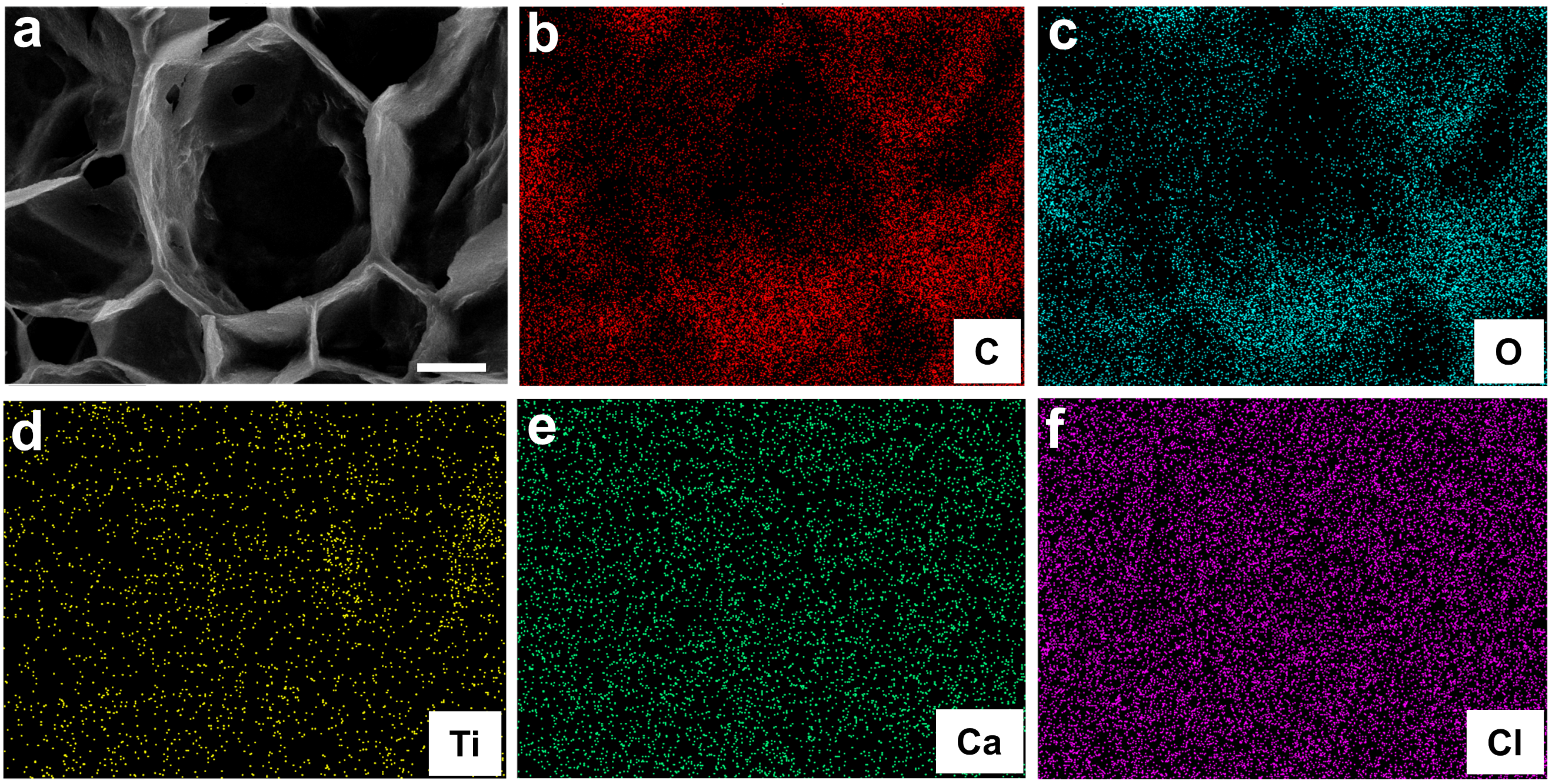


**Fig. S11** (**a**) SEM image of SF/CNTs/MXene (SCM)_(O/W)_ hydrogel and the corresponding (**b**) C, (**c**) O, (**d**) Ti, (**e**) Ca, and (**f**) Cl element mappings. Scale bar, 10 μm


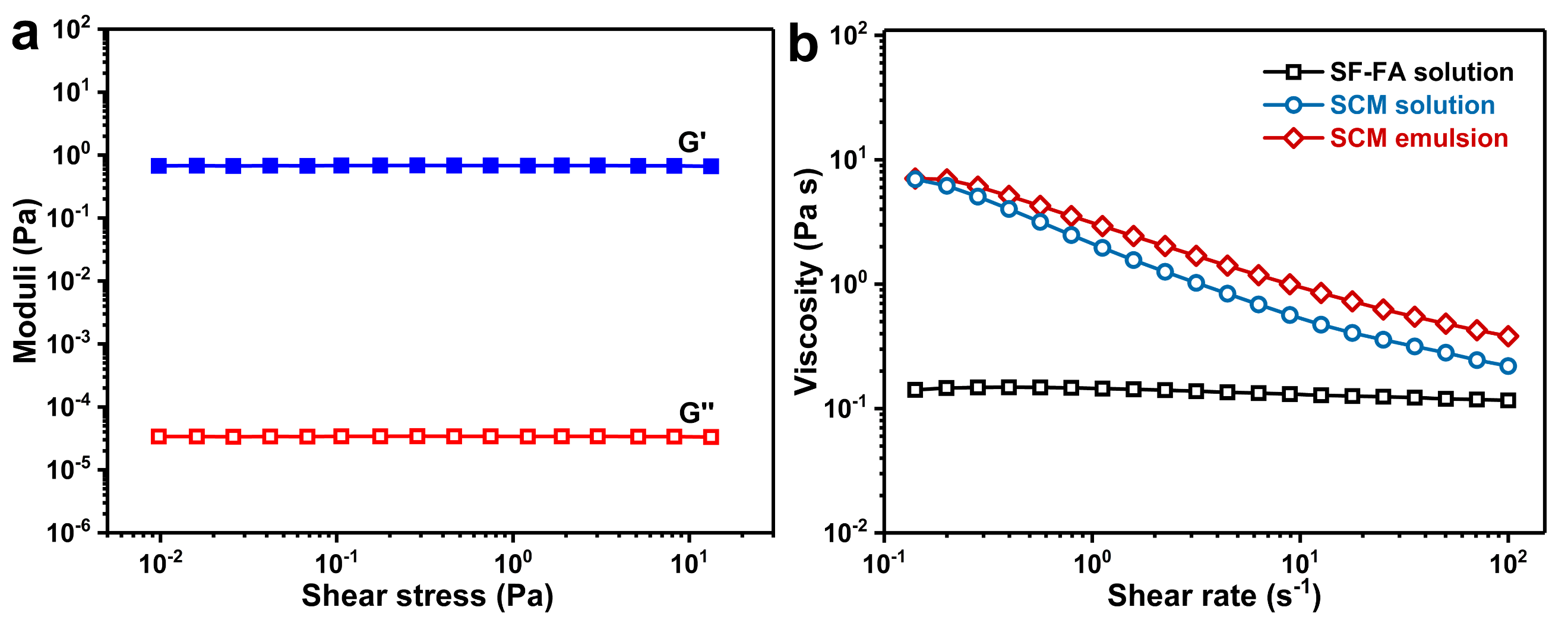


**Fig. S12** (**a**) The moduli (G′, G′′) of SF/LiCl-FA solition. (**b**) The viscosities of SCM solution and emulsion with different shear rates


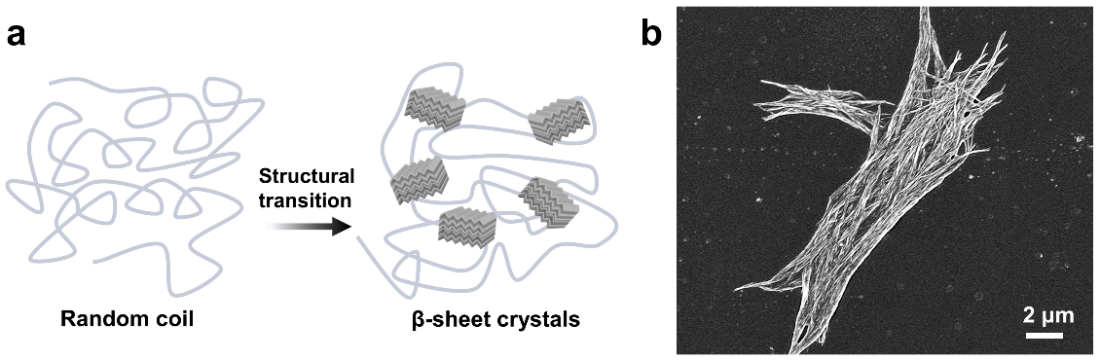


**Fig. S13** (**a**) The secondary structural transition within silk fibroin. (**b**) SEM image of silk fibroin fibrils


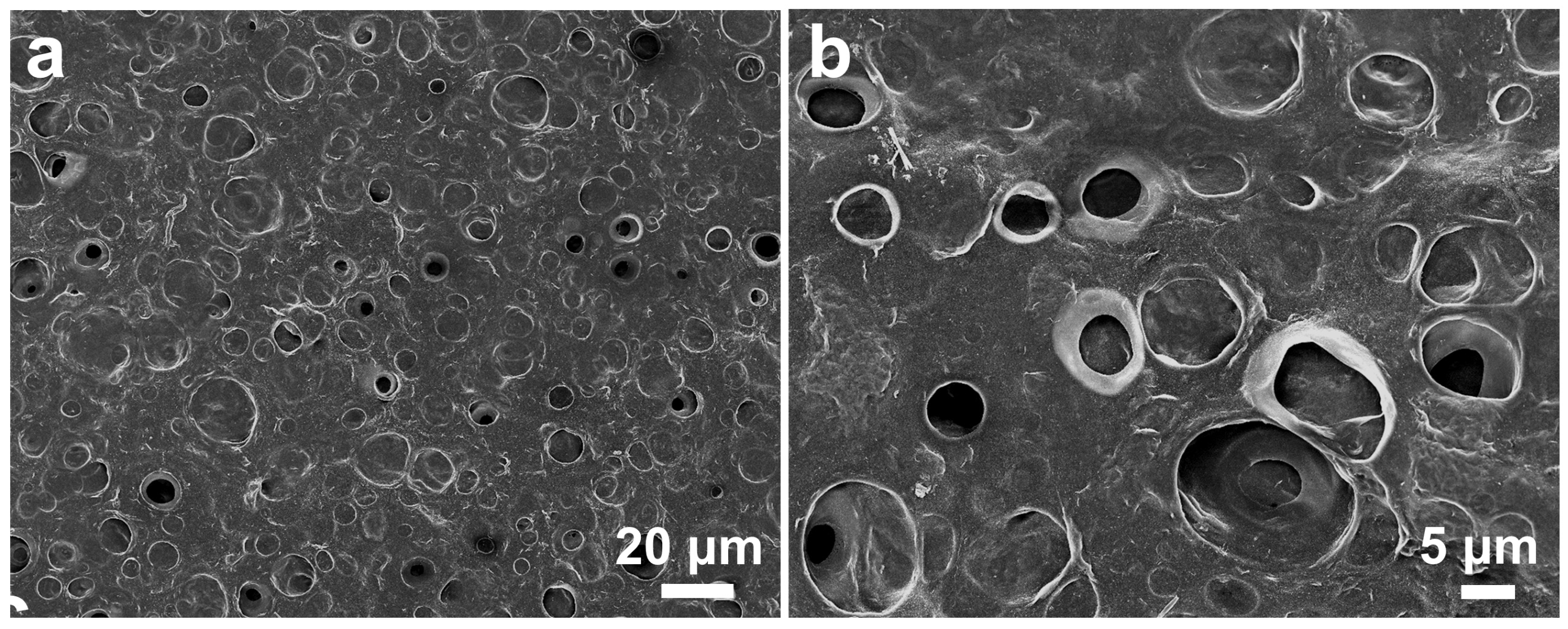


**Fig. S14** SEM images of SCM_(O/W)_ hydrogel surface at (**a**) low and (**b**) high magnifications


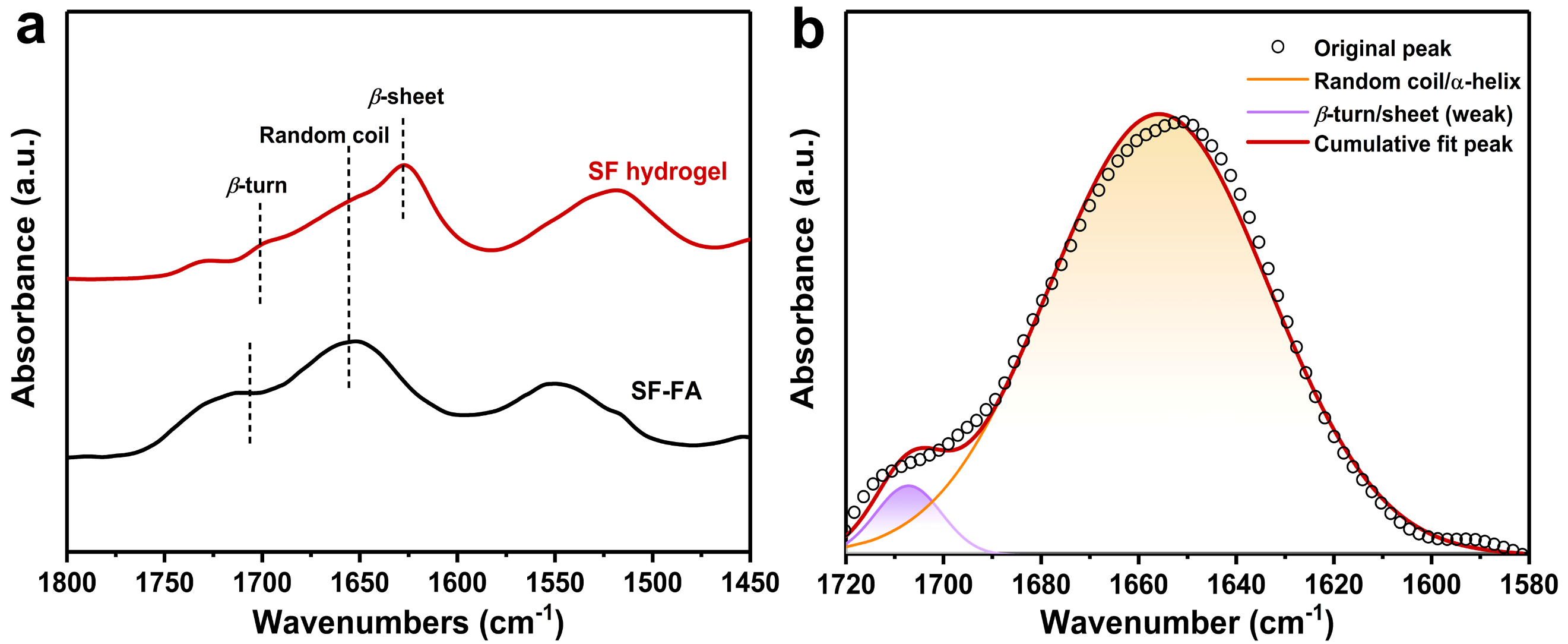


**Fig. S15** (**a**) FT-IR spectra in amide I band of SF-FA solution and RSF hydrogel. (**b**) The deconvolution in amide I band of SF-FA solution


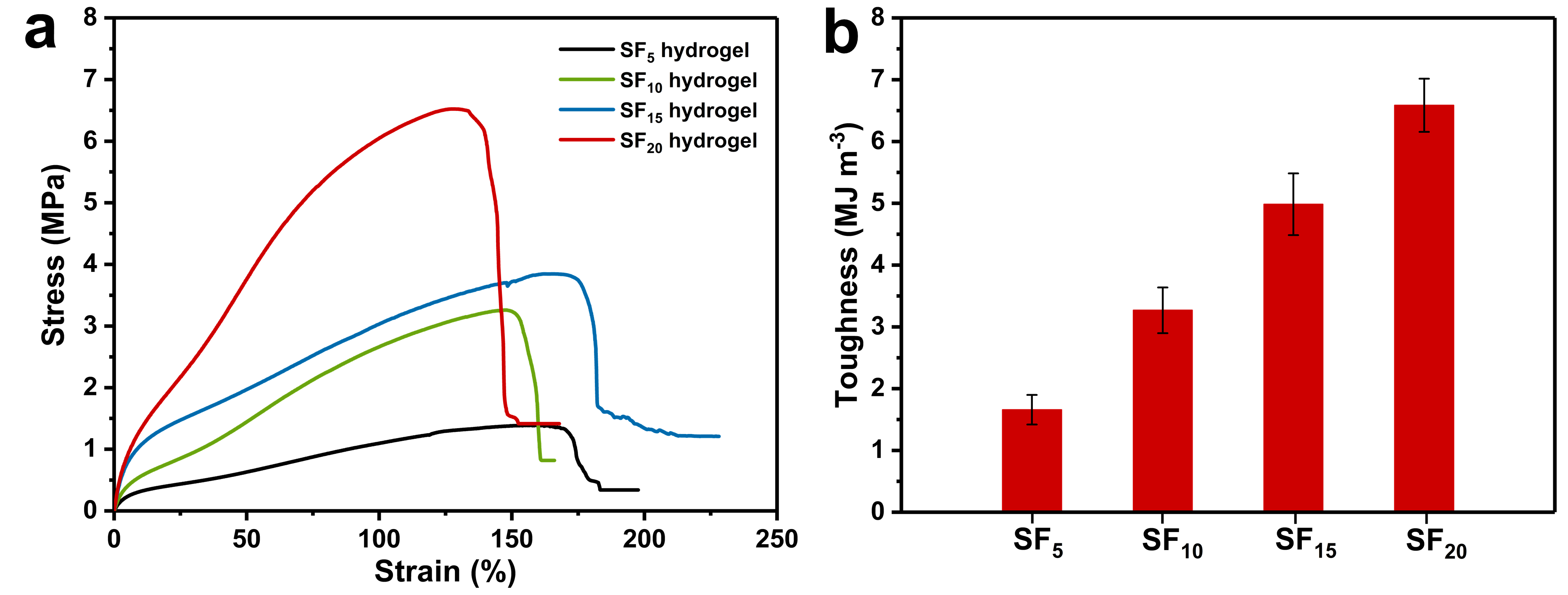


**Fig. S16** (**a**) Typical tensile stress-strain curves and (**b**) and toughness of SF hydrogels with different mass fractions


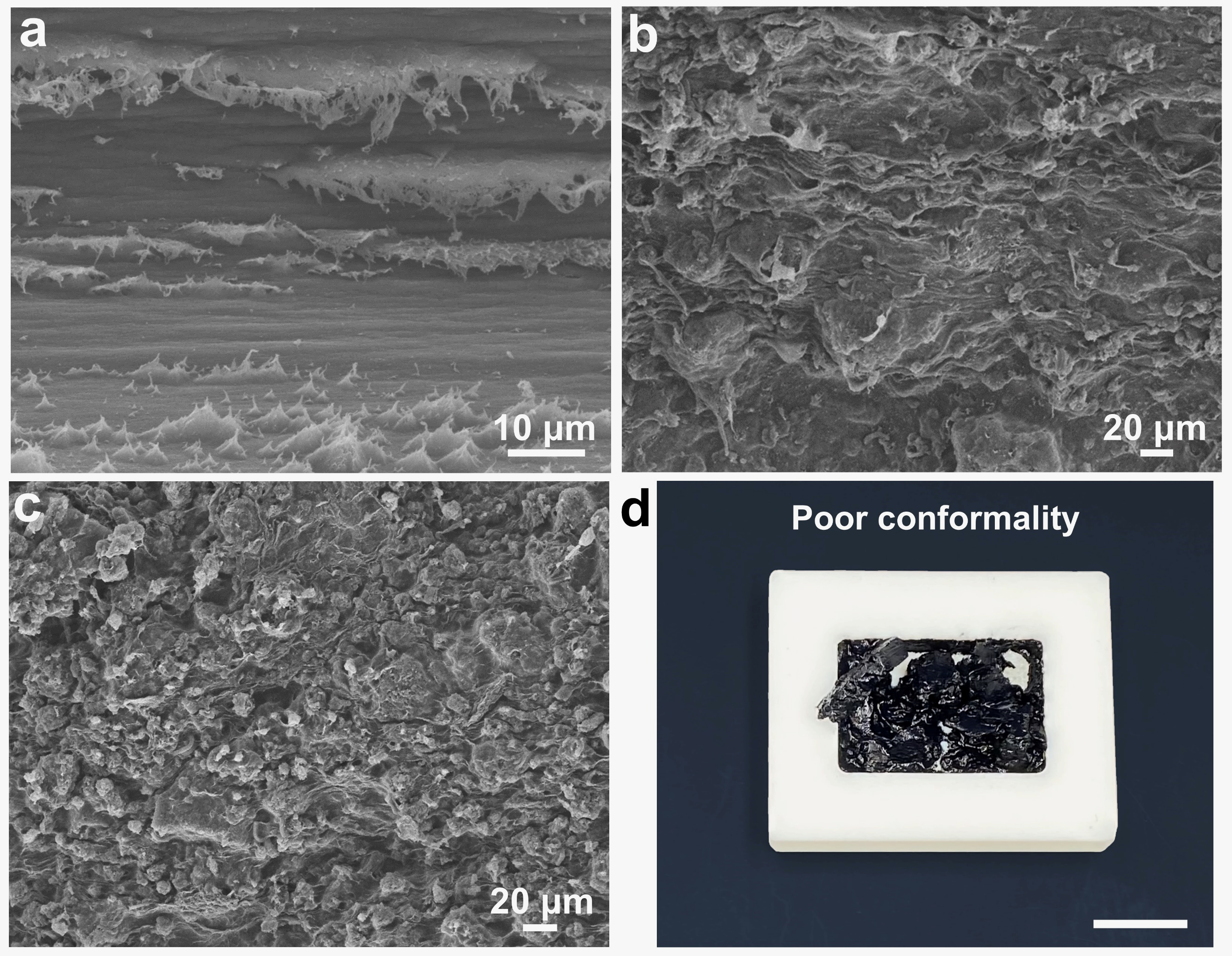


**Fig. S17** Cross-sectional SEM images of (**a**) SF_10_, (**b**) SF_10_F_10_, and (**c**) SF_10_F_50_ hydrogels after pulling. (**d**) The poor shape stability of SCM hydrogel when the filling exceeds 50 wt%. Scale bar, 20 mm


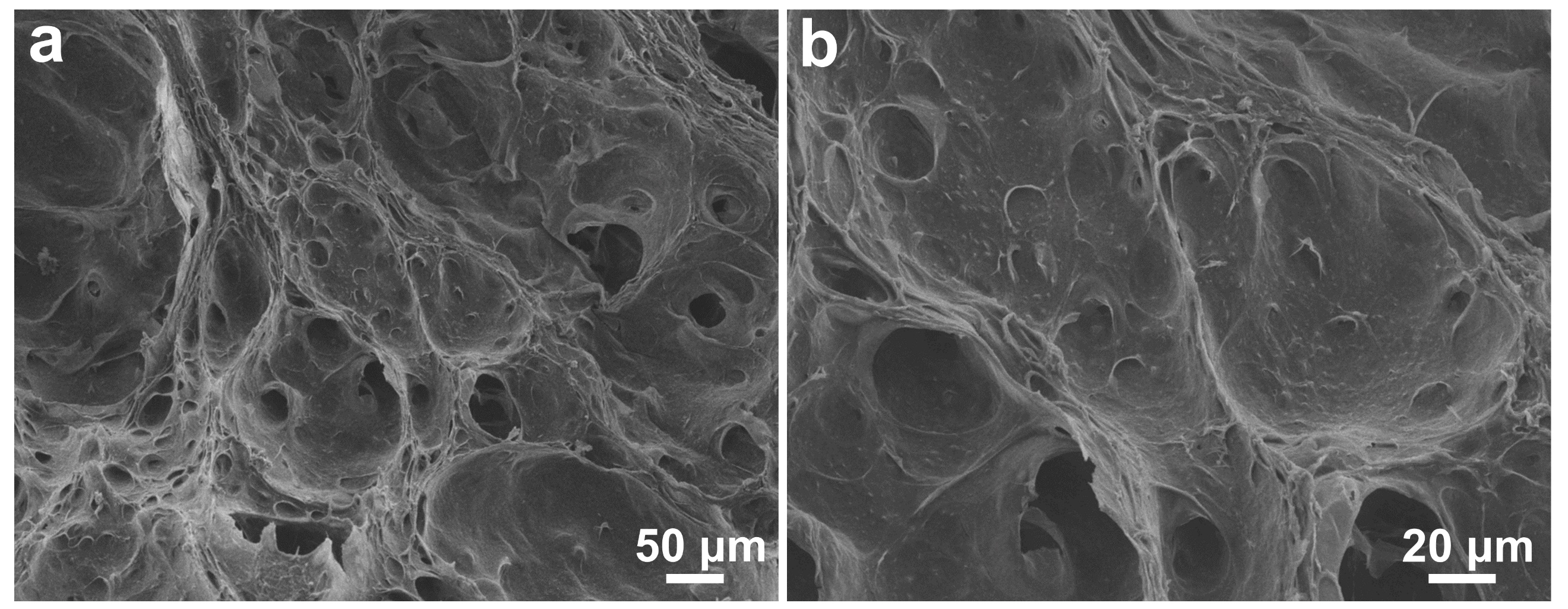


**Fig. S18** Cross-sectional SEM images of SF_10_F_50_ (O/W) hydrogel after tensile fracture at (**a**) low and (**b**) high magnifications


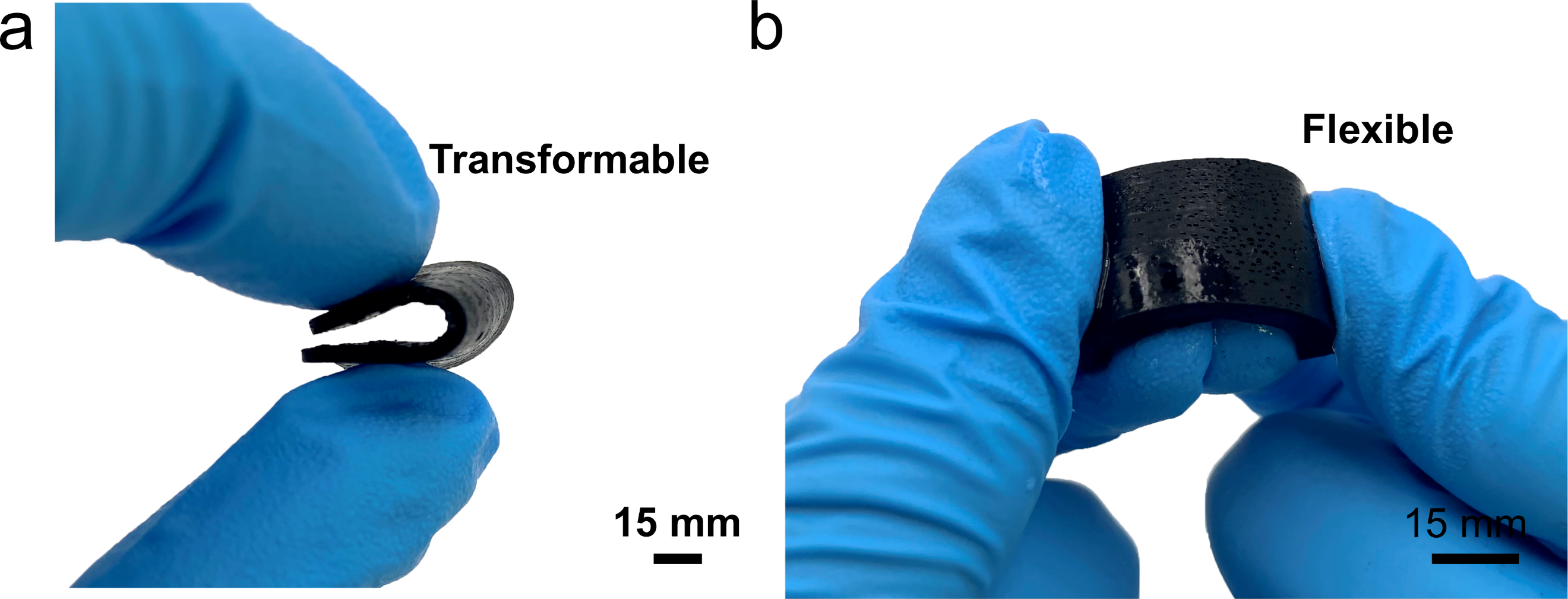


**Fig. S19** (**a**) deformability and (**b**) flexibility of SF_10_F_30_ (O/W) hydrogels


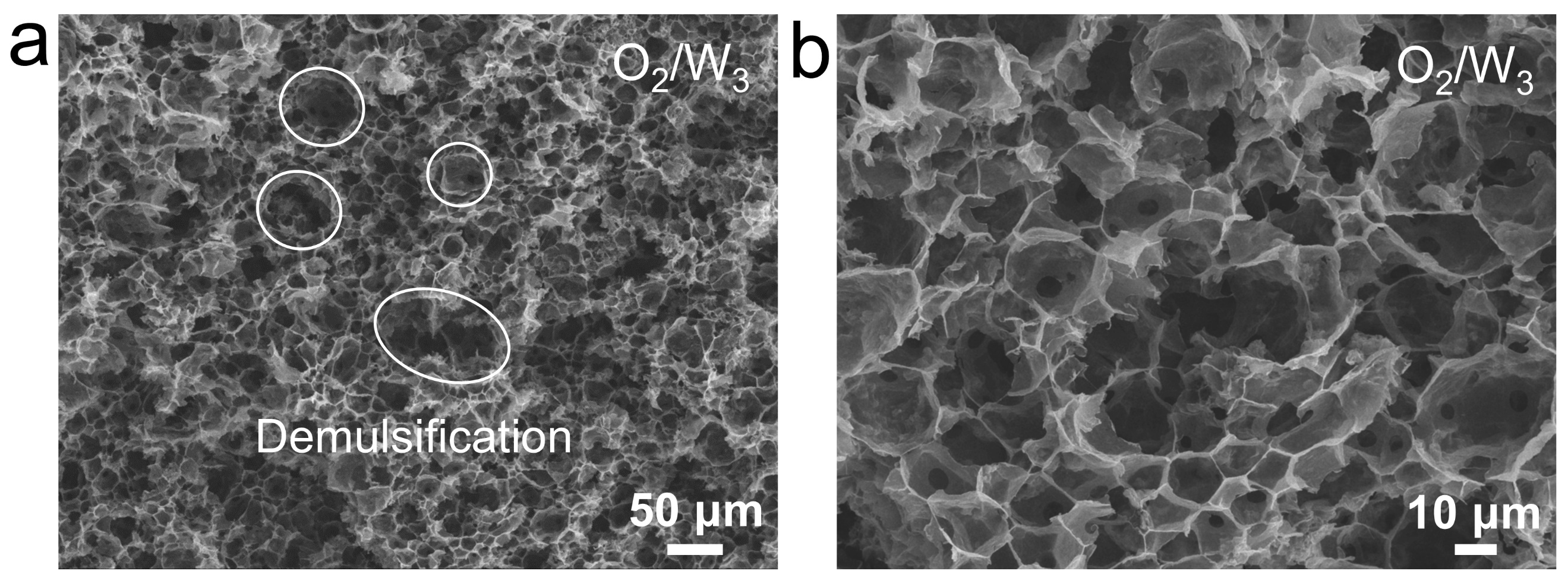


**Fig. S20** SEM images of SCM_(O2/W3)_ hydrogels at (**a**) low and (**b**) high magnifications. The white circles represent the broken aperture walls


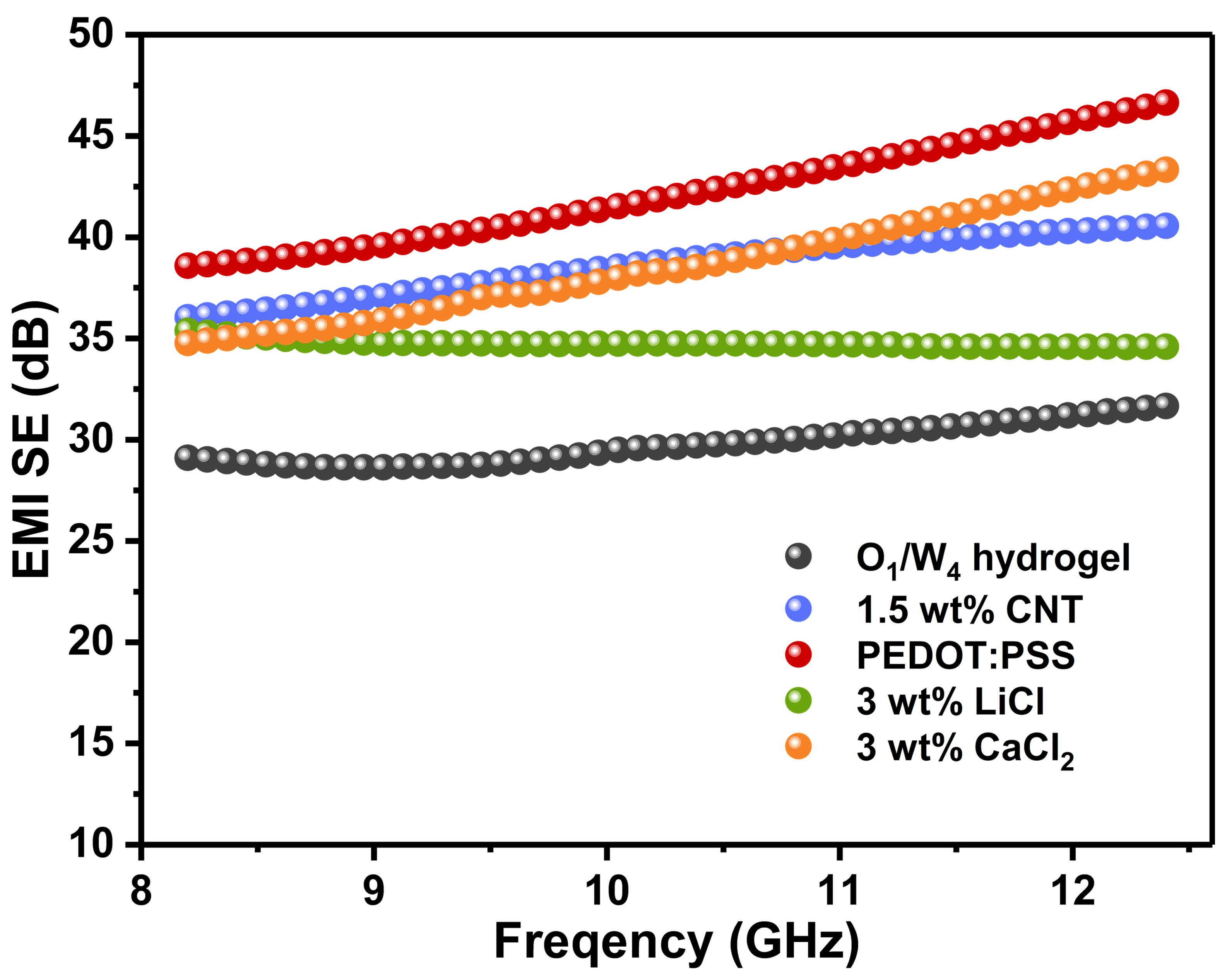


**Fig. S21** EMI SE curves of different assembled hydrogels


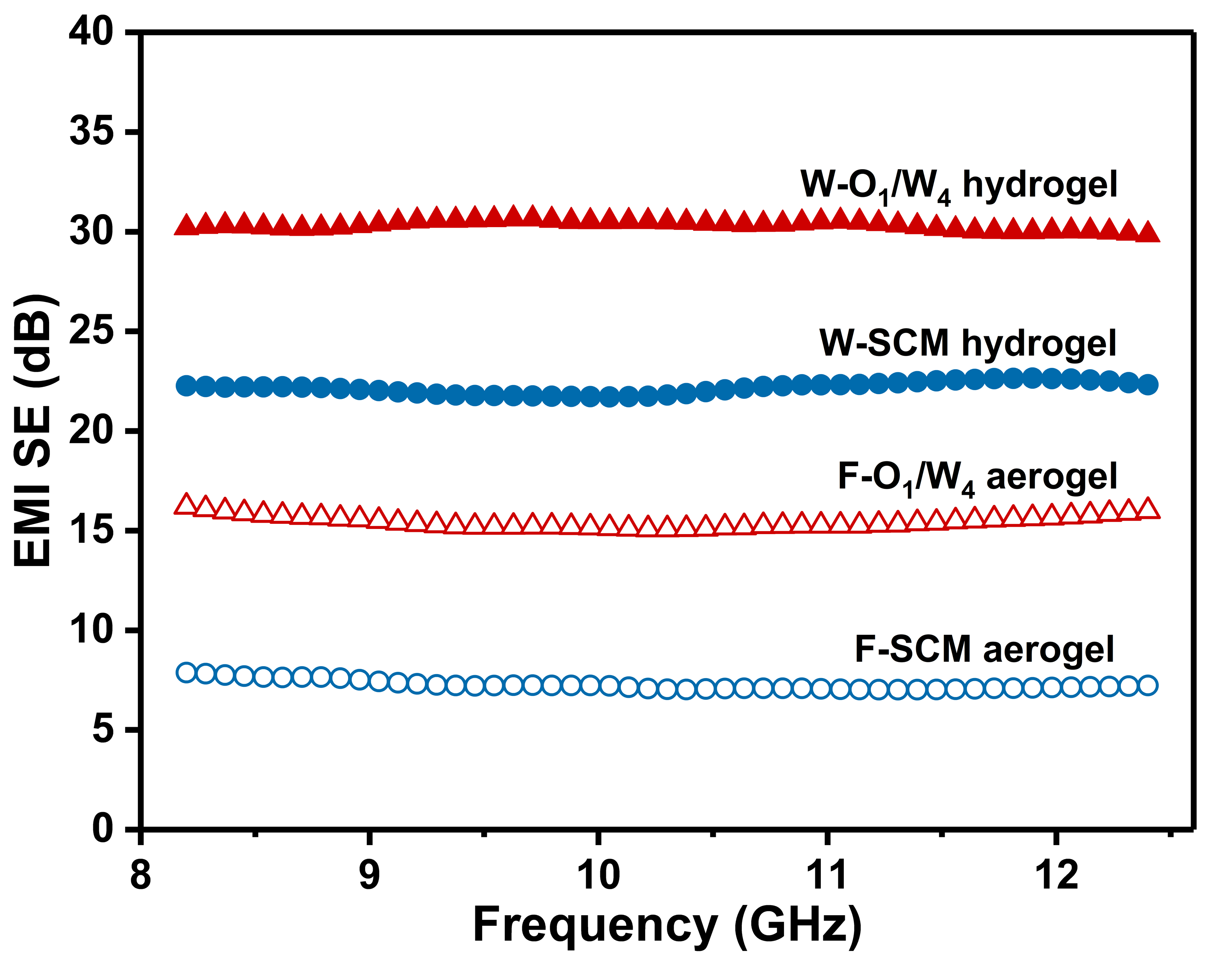


**Fig. S22** EMI SE curves of SCM and SCM_(O1/W4)_ samples in the frequency range of 8-12 GHz (X band)


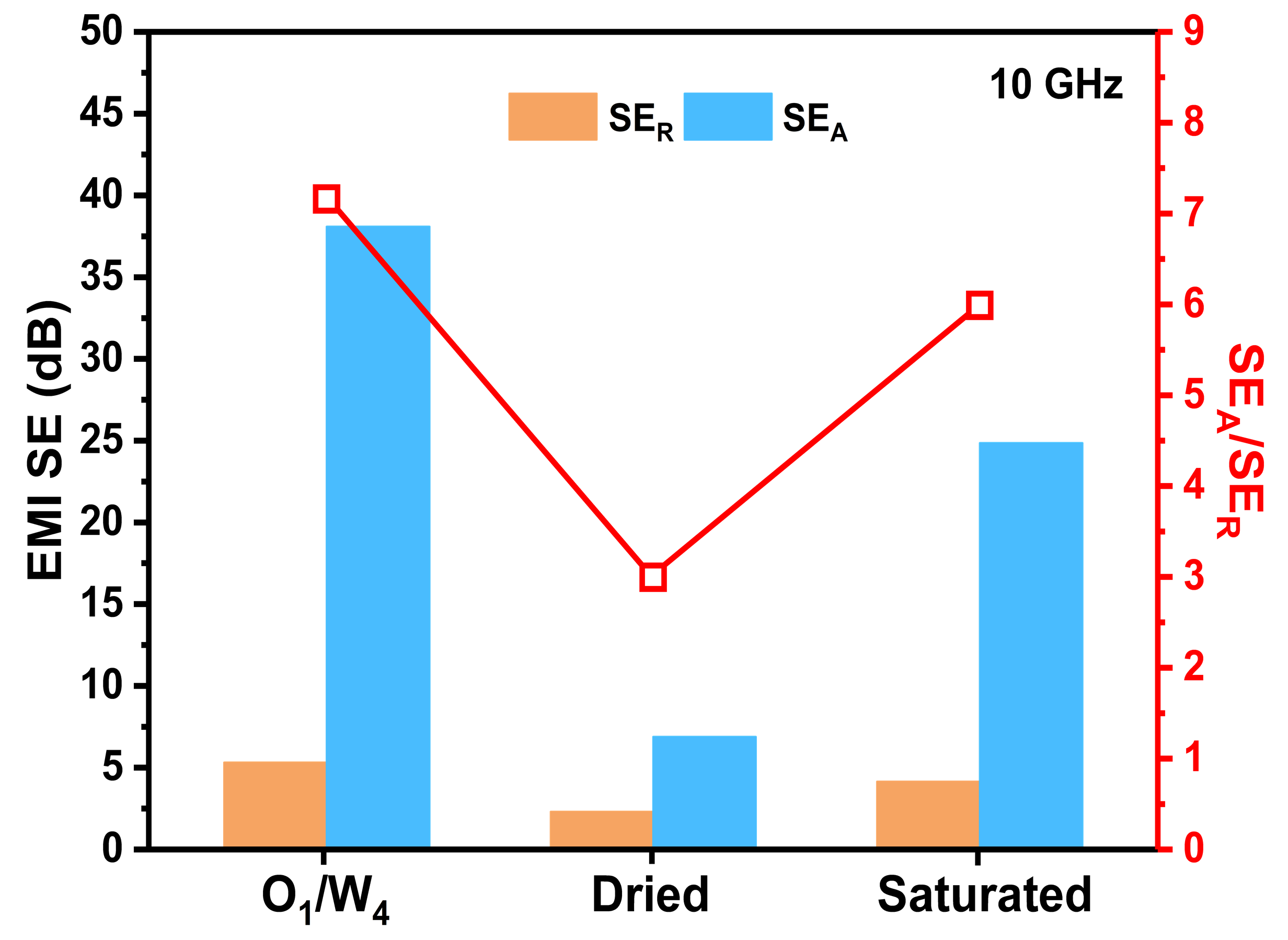


**Fig. S23** Comparison of SE_Total_, SE_A_, and SE_R_ of SCM_(O1/W4)_ samples in different states


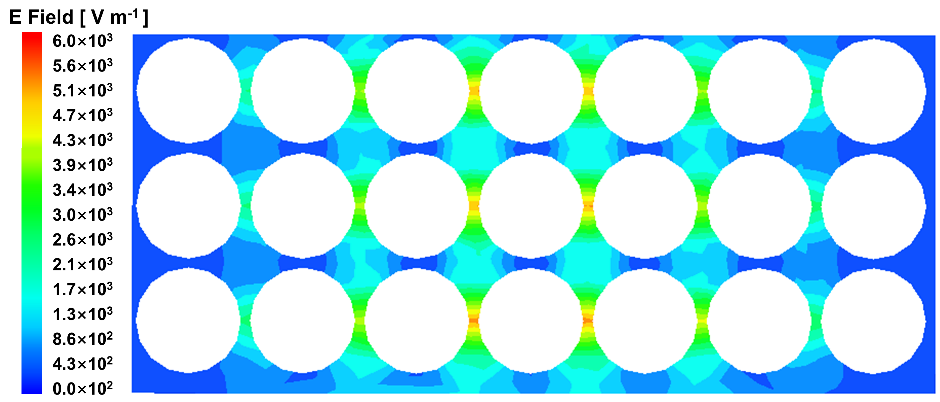


**Fig. S24** Simulated energy distribution of electric field for deionized water


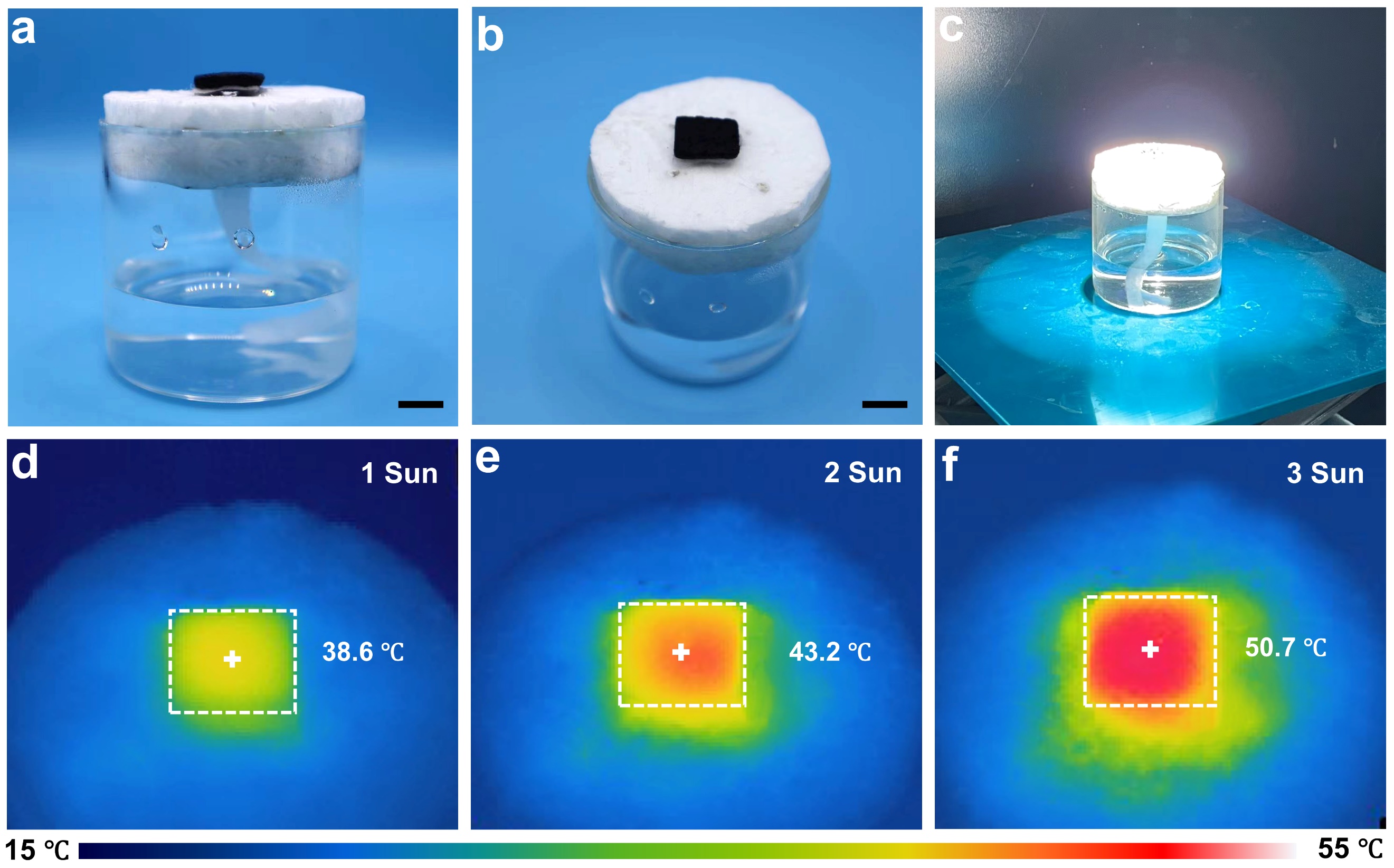


**Fig. S25** (**a-c**) The digital photographs of hydrogel generator and (**d-f**) thermal images under 1-, 2-, and 3- sun irradiation, respectively. Scale bar, 30 mm


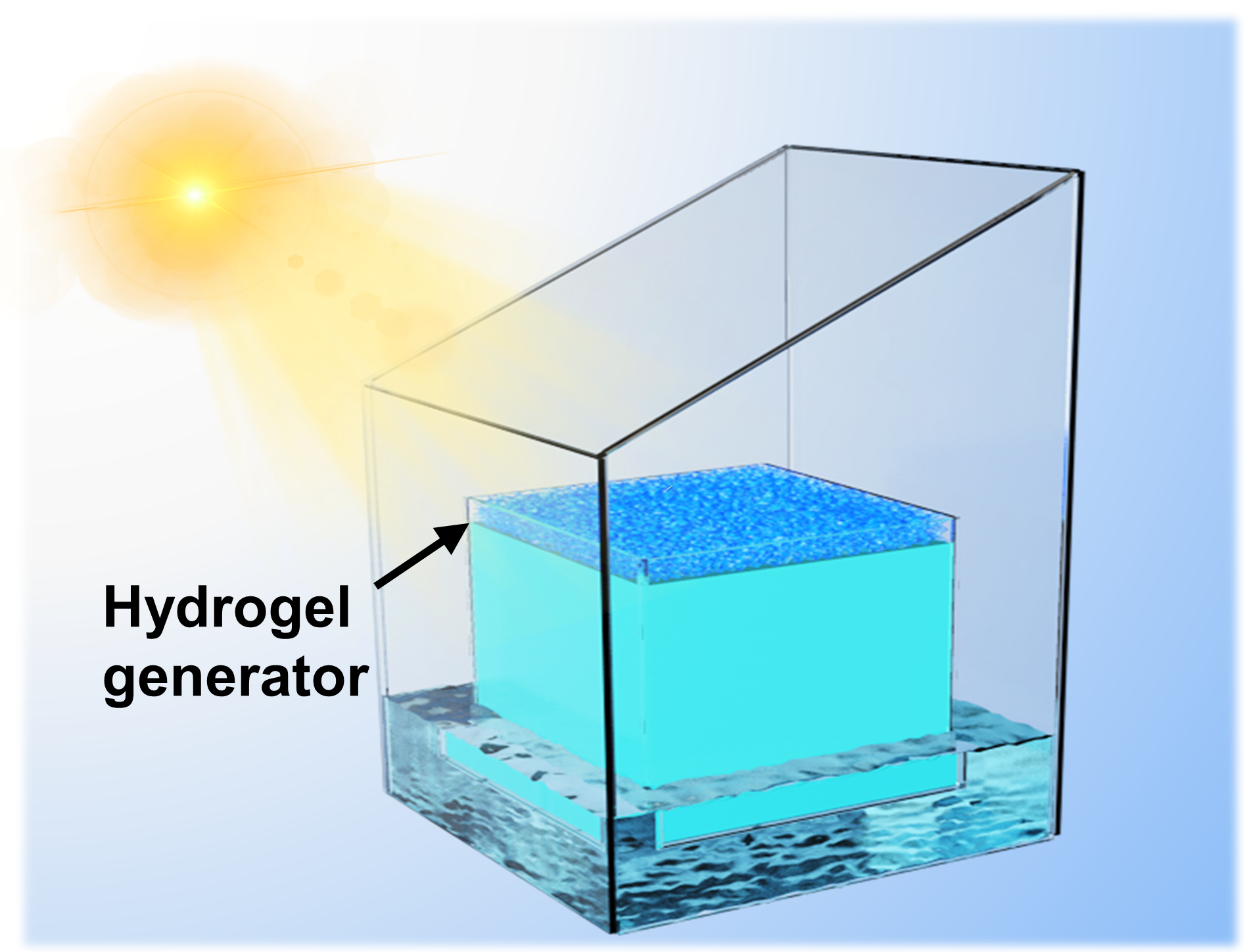


**Fig. S26** Solar-driven water evaporation device for hydrogel generator


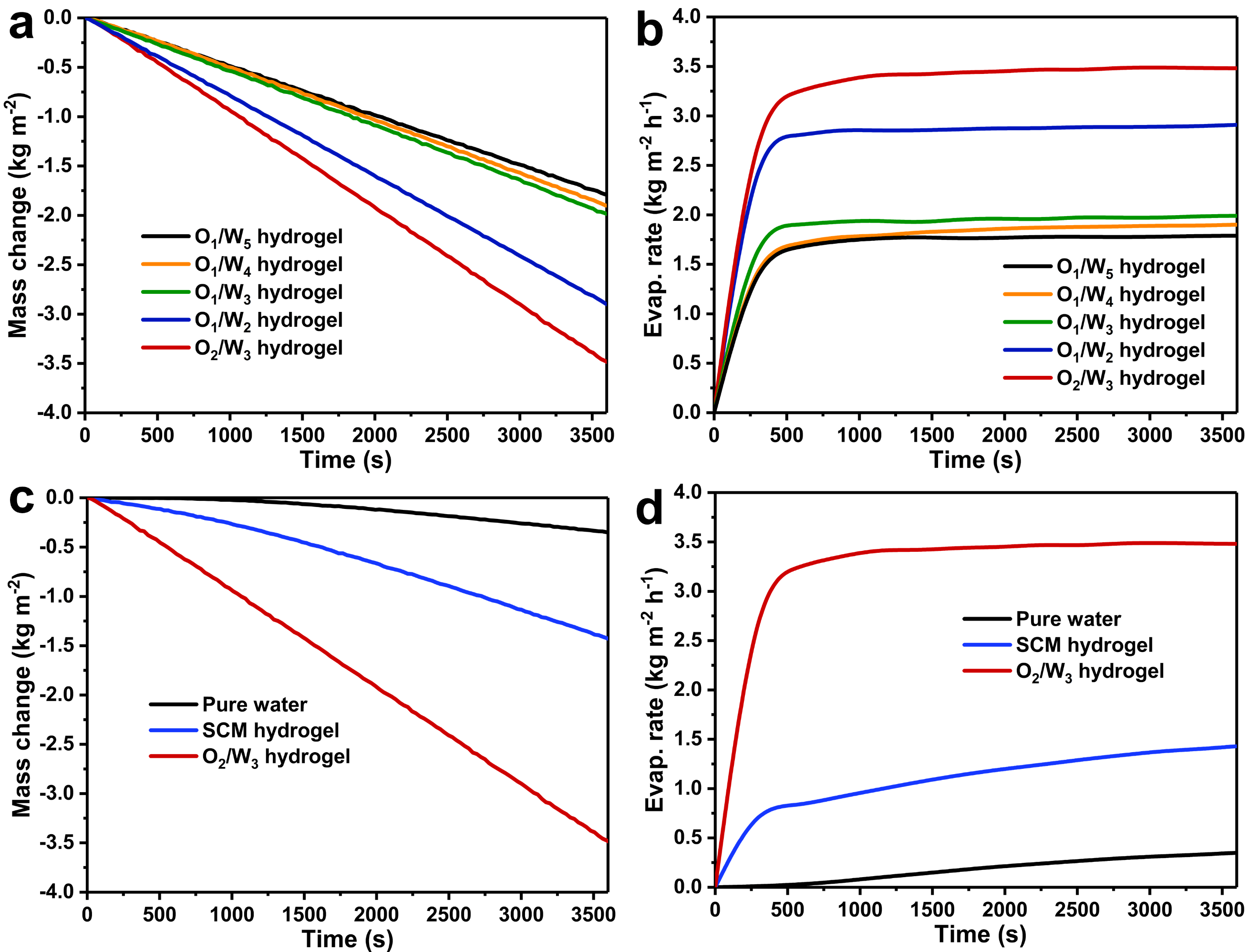


**Fig. S27** (**a**) Mass changes and (**b**) water vaporation rates of different evaporators under 1 sun irradiation. (**c-d**) Comparison of solar-driven water evaporation properties of SCM and SCM_(O2/W3)_ hydrogel generators





**Fig. S28** Comparison of the water evaporation rates and energy efficiencies with the reported materials under 1-sun irradiation


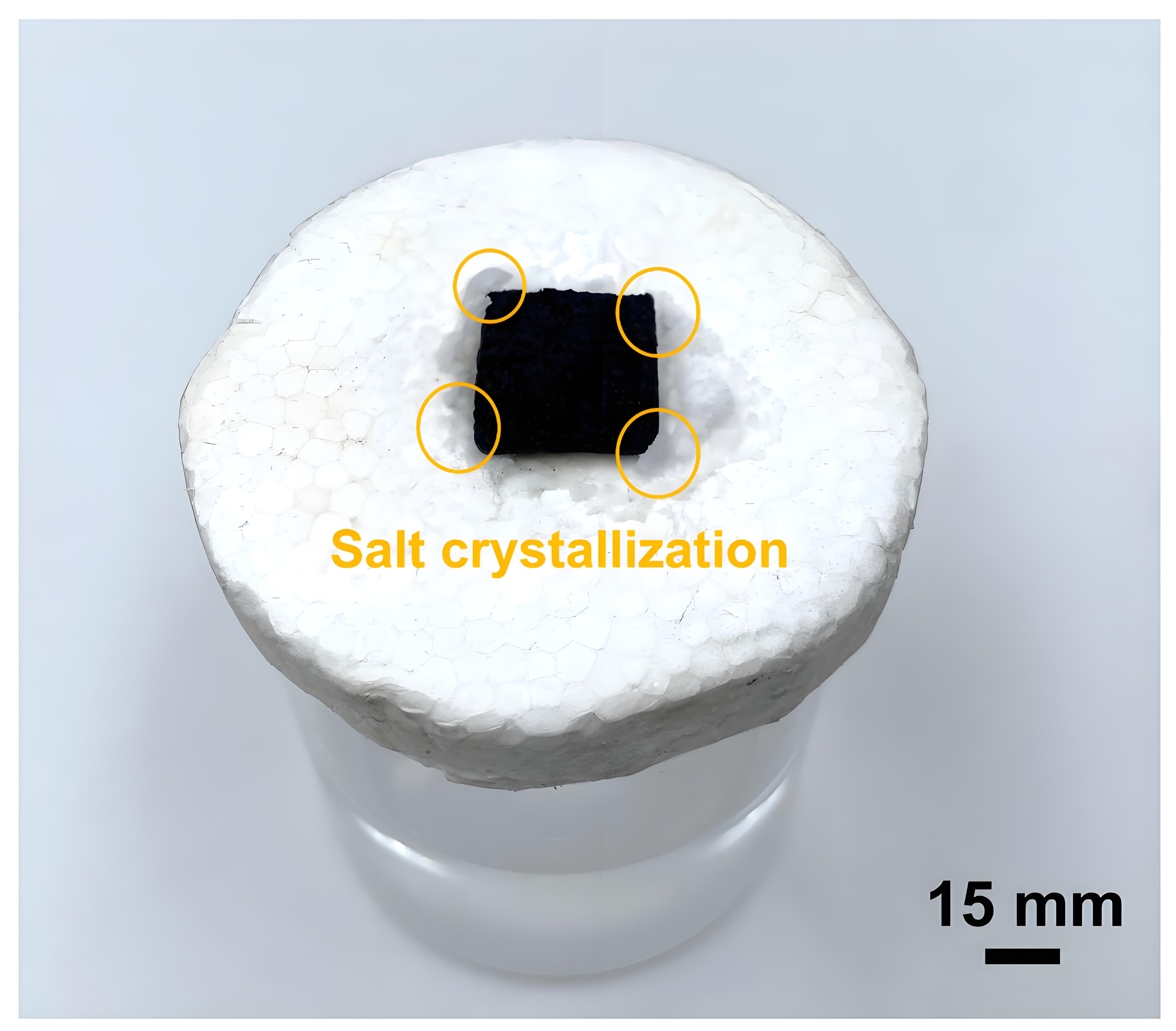


**Fig. S29** Salt crystallization after 60 h of solar-driven brine evaporation

**Table S1** Comparison of EMI shielding performance of polymer-based EMI shielding materials

| **Entry** | **Materials** | **EMI SE (dB)** | **Thickness (mm)** | **EMI SE/thickness**  **（dB/mm）** | **References** |
| --- | --- | --- | --- | --- | --- |
| **1** | **Hydrogel evaporator (O_1_/W_4_)** | **44** | **1.5** | **29.3** | **This work** |
| 2 | TAPU/Fe_3_O_4_@PANI hydrogel | 26 | 2 | 13 | [S1] |
| 3 | PMG organohydrogel | 41 | 2 | 20.5 | [S2] |
| 4 | MXene-based nanocomposite | 55.2 | 5 | 11.04 | [S3] |
| 5 | RG hydrogels | 26.5 | 2 | 13.25 | [S4] |
| 6 | Graphene foam/PDMS | 30 | 2 | 15 | [S5] |
| 7 | rGO/PS composite | 45.1 | 2.5 | 18.04 | [S6] |
| 8 | CNT/PP composite | 48.3 | 2.2 | 22 | [S7] |
| 9 | MWCNT/WPU foam | 50 | 2.3 | 21.7 | [S8] |
| 10 | CNT sponge/epoxy | 25 | 1.5 | 16.7 | [S9] |
| 11 | CNT/PLLA foam | 20.3 | 2.5 | 8.12 | [S10] |
| 12 | PLA/CNC/CNT | 31 | 1.5 | 20.67 | [S11] |
| 13 | EGaIn/Silicone | 55.7 | 3 | 18.6 | [S12] |
| 14 | PVA/CS/MXene hydrogel | 30.7 | 3 | 10.23 | [S13] |
| 15 | PVA/MXene hydrogel | 59 | 4 | 14.75 | [S14] |

**Table S2** Comparison of solar vapor generation performance of hydrogel evaporator (O_2_/W_3_) with previous photothermal hydrogel materials under 1 kW m^−2^ irradiation

| **Entry** | **Materials** | **Evaporation rate**  **(kg m^-2^ h^-1^)** | **Efficiency**  **(%)** | **References** |
| --- | --- | --- | --- | --- |
| **1** | **Hydrogel evaporator (O_2_/W_3_)** | **3.50** | **92.2** | **This work** |
| 2 | P-CHG hydrogel | 3.02 | 89.1 | [S15] |
| 3 | HHE hydrogel | 3.20 | 90.0 | [S16] |
| 4 | HNG hydrogel | 3.20 | 94.0 | [S17] |
| 5 | CTH hydrogel | 2.50 | 95.0 | [S18] |
| 6 | Hy-P-CW hydrogel | 1.92 | 90.7 | [S19] |
| 7 | HBA-NCS hydrogel | 2.03 | 96.3 | [S20] |
| 8 | C-ADM hydrogel | 2.74 | 91.6 | [S21] |
| 9 | CVM4 hydrogel | 2.33 | 84.7 | [S22] |
| 10 | APH hydrogel | 2.50 | 90.7 | [S23] |
| 11 | LC-LCG hydrogel | 1.84 | 86.5 | [S24] |
| 12 | DSH hydrogel | 2.60 | 91.0 | [S25] |
| 13 | PSBMA hydrogel | 2.02 | 97.5 | [S26] |
| 14 | PPy-PEI-A-CNF hydrogel | 1.66 | 94.6 | [S27] |
| 15 | CACH hydrogel | 1.33 | 90.6 | [S28] |
| 16 | HPH hydrogel | 1.67 | 83.2 | [S29] |
| 17 | CPHs hydrogel | 1.59 | 89.5 | [S30] |
| 18 | SHAF hydrogel | 2.20 | 93.5 | [S31] |
| 19 | COF hydrogel | 2.50 | 93.2 | [S32] |
| 20 | Pvbips/PPy Hydrogel | 3.17 | 87.8 | [S33] |

**Supplementary References**

1. Y. Liu, Z. Zhang, X. Yang, F. Li, Z. Liang et al., A stretchable, environmentally stable, and mechanically robust nanocomposite polyurethane organohydrogel with anti-freezing, anti-dehydration, and electromagnetic shielding properties for strain sensors and magnetic actuators. J. Mater. Chem. A **11**(12), 6603–6614 (2023). <https://doi.org/10.1039/D2TA09205K>
2. J. Fang, J. Xu, P. Zuo, Y. Zhou, C. Tang et al., New system for green EMI shielding: organohydrogel with multi-band green electromagnetic shielding, sensing, and infrared-stealth capacity. J. Mater. Sci. Technol. **219**, 1–9 (2025). <https://doi.org/10.1016/j.jmst.2024.10.005>
3. G.-C. Xu, Y. Nie, H.-N. Li, W.-L. Li, W.-T. Lin et al., Supergravity-steered generic manufacturing of nanosheets-embedded nanocomposite hydrogel with highly oriented, heterogeneous architecture. Adv. Mater. **36**(24), 2400075 (2024). <https://doi.org/10.1002/adma.202400075>
4. R. Yin, C. Zhang, Y. Chen, Y. Wang, Q. Feng et al., Transient, printable and recyclable gelatin hydrogels with enhanced mechanical sensing and electromagnetic shielding performance by incorporation of reduced graphene oxide. Chem. Eng. J. **475**, 145794 (2023). <https://doi.org/10.1016/j.cej.2023.145794>
5. Z. Chen, C. Xu, C. Ma, W. Ren, H.-M. Cheng, Lightweight and flexible graphene foam composites for high-performance electromagnetic interference shielding. Adv. Mater. **25**(9), 1296–1300 (2013). <https://doi.org/10.1002/adma.201204196>
6. D.-X. Yan, H. Pang, B. Li, R. Vajtai, L. Xu et al., Structured reduced graphene oxide/polymer composites for ultra-efficient electromagnetic interference shielding. Adv. Funct. Mater. **25**(4), 559–566 (2015). <https://doi.org/10.1002/adfm.201403809>
7. H.-Y. Wu, L.-C. Jia, D.-X. Yan, J.-F. Gao, X.-P. Zhang et al., Simultaneously improved electromagnetic interference shielding and mechanical performance of segregated carbon nanotube/polypropylene composite *via* solid phase molding. Compos. Sci. Technol. **156**, 87–94 (2018). <https://doi.org/10.1016/j.compscitech.2017.12.027>
8. Z. Zeng, H. Jin, M. Chen, W. Li, L. Zhou et al., Lightweight and anisotropic porous MWCNT/WPU composites for ultrahigh performance electromagnetic interference shielding. Adv. Funct. Mater. **26**(2), 303–310 (2016). <https://doi.org/10.1002/adfm.201503579>
9. Y. Chen, H.-B. Zhang, Y. Yang, M. Wang, A. Cao et al., High-performance epoxy nanocomposites reinforced with three-dimensional carbon nanotube sponge for electromagnetic interference shielding. Adv. Funct. Mater. **26**(3), 447–455 (2016). <https://doi.org/10.1002/adfm.201503782>
10. T. Kuang, L. Chang, F. Chen, Y. Sheng, D. Fu et al., Facile preparation of lightweight high-strength biodegradable polymer/multi-walled carbon nanotubes nanocomposite foams for electromagnetic interference shielding. Carbon **105**, 305–313 (2016). <https://doi.org/10.1016/j.carbon.2016.04.052>
11. B. Yu, Z. Zhao, S. Fu, L. Meng, Y. Liu et al., Fabrication of PLA/CNC/CNT conductive composites for high electromagnetic interference shielding based on Pickering emulsions method. Compos. Part A Appl. Sci. Manuf. **125**, 105558 (2019). <https://doi.org/10.1016/j.compositesa.2019.105558>
12. B. Yao, W. Hong, T. Chen, Z. Han, X. Xu et al., Highly stretchable polymer composite with strain-enhanced electromagnetic interference shielding effectiveness. Adv. Mater. **32**(14), e1907499 (2020). <https://doi.org/10.1002/adma.201907499>
13. C. Wang, L. Xu, J. Zheng, Z. Zhu, Z. Huang et al., Polyvinyl alcohol/chitosan biomimetic hydrogel enhanced by MXene for excellent electromagnetic shielding and pressure sensing. Int. J. Biol. Macromol. **278**, 134354 (2024). <https://doi.org/10.1016/j.ijbiomac.2024.134354>
14. C. Wang, Z. Zhu, L. Han, L. Xu, M. Wang et al., Poly(vinyl alcohol) hydrogels enhanced with Ti_3_C_2_T*_x_* MXene nanosheets and aramid nanofibers for electromagnetic shielding and motion detection. ACS Appl. Nano Mater. **7**(21), 24925–24937 (2024). <https://doi.org/10.1021/acsanm.4c04840>
15. L. Shu, X.-F. Zhang, Z. Wang, J. Liu, J. Yao, Cellulose-based bi-layer hydrogel evaporator with a low evaporation enthalpy for efficient solar desalination. Carbohydr. Polym. **327**, 121695 (2024). <https://doi.org/10.1016/j.carbpol.2023.121695>
16. Y. Guo, H. Lu, F. Zhao, X. Zhou, W. Shi et al., Biomass-derived hybrid hydrogel evaporators for cost-effective solar water purification. Adv. Mater. **32**(11), e1907061 (2020). <https://doi.org/10.1002/adma.201907061>
17. F. Zhao, X. Zhou, Y. Shi, X. Qian, M. Alexander et al., Highly efficient solar vapour generation *via* hierarchically nanostructured gels. Nat. Nanotechnol. **13**(6), 489–495 (2018). <https://doi.org/10.1038/s41565-018-0097-z>
18. X. Zhou, F. Zhao, Y. Guo, Y. Zhang, G. Yu, A hydrogel-based antifouling solar evaporator for highly efficient water desalination. Energy Environ. Sci. **11**(8), 1985–1992 (2018). <https://doi.org/10.1039/c8ee00567b>
19. D. Fan, Y. Lu, X. Xu, Y. Tang, H. Zhang et al., Multifunctional wood-based hydrogels for wastewater treatment and interfacial solar steam generation. Chem. Eng. J. **471**, 144421 (2023). <https://doi.org/10.1016/j.cej.2023.144421>
20. C. Song, M.S. Irshad, Z. Li, J. Hu, G. Shao et al., Enhancing solar steam generation in hydrogel evaporator by bio-based microfluidic component. Chem. Eng. J. **478**, 146566 (2023). <https://doi.org/10.1016/j.cej.2023.146566>
21. H. Li, G. Tong, A. Chu, J. Chen, H. Yang et al., Thermoresponsive Janus hybrid hydrogel for efficient solar steam generation. Nano Energy **124**, 109475 (2024). <https://doi.org/10.1016/j.nanoen.2024.109475>
22. A. Chu, M. Yang, H. Yang, X. Shi, J. Chen et al., Sustainable self-cleaning evaporators for highly efficient solar desalination using a highly elastic sponge-like hydrogel. ACS Appl. Mater. Interfaces **14**(31), 36116–36131 (2022). <https://doi.org/10.1021/acsami.2c08561>
23. C. Li, B. Zhu, Z. Liu, J. Zhao, R. Meng et al., Polyelectrolyte-based photothermal hydrogel with low evaporation enthalpy for solar-driven salt-tolerant desalination. Chem. Eng. J. **431**, 134224 (2022). <https://doi.org/10.1016/j.cej.2021.134224>
24. X. Lin, P. Wang, R. Hong, X. Zhu, Y. Liu et al., Fully lignocellulosic biomass-based double-layered porous hydrogel for efficient solar steam generation. Adv. Funct. Mater. **32**(51), 2209262 (2022). <https://doi.org/10.1002/adfm.202209262>
25. Y. Guo, F. Zhao, X. Zhou, Z. Chen, G. Yu, Tailoring nanoscale surface topography of hydrogel for efficient solar vapor generation. Nano Lett. **19**(4), 2530–2536 (2019). <https://doi.org/10.1021/acs.nanolett.9b00252>
26. B. Peng, Q. Lyu, M. Li, S. Du, J. Zhu et al., Phase-separated polyzwitterionic hydrogels with tunable sponge-like structures for stable solar steam generation. Adv. Funct. Mater. **33**(18), 2214045 (2023). <https://doi.org/10.1002/adfm.202214045>
27. R. Zhu, D. Wang, J. Xie, Y. Liu, M. Liu et al., Salt-resistant Schiff base cross-linked superelastic photothermal cellulose aerogels for long-term seawater desalination. Chem. Eng. J. **427**, 131618 (2022). <https://doi.org/10.1016/j.cej.2021.131618>
28. J. Yuan, X. Lei, C. Yi, H. Jiang, F. Liu et al., 3D-printed hierarchical porous cellulose/alginate/carbon black hydrogel for high-efficiency solar steam generation. Chem. Eng. J. **430**, 132765 (2022). <https://doi.org/10.1016/j.cej.2021.132765>
29. F. Zhu, L. Wang, B. Demir, M. An, Z.L. Wu et al., Accelerating solar desalination in brine through ion activated hierarchically porous polyion complex hydrogels. Mater. Horiz. **7**(12), 3187–3195 (2020). <https://doi.org/10.1039/D0MH01259A>
30. B. Peng, Y. Gao, Q. Lyu, Z. Xie, M. Li et al., Cationic photothermal hydrogels with bacteria-inhibiting capability for freshwater production *via* solar-driven steam generation. ACS Appl. Mater. Interfaces **13**(31), 37724–37733 (2021). <https://doi.org/10.1021/acsami.1c10854>
31. C. Wen, H. Guo, J. Yang, Q. Li, X. Zhang et al., Zwitterionic hydrogel coated superhydrophilic hierarchical antifouling floater enables unimpeded interfacial steam generation and multi-contamination resistance in complex conditions. Chem. Eng. J. **421**, 130344 (2021). <https://doi.org/10.1016/j.cej.2021.130344>
32. Z. Huang, Y.-H. Luo, W.-Y. Geng, Y. Wan, S. Li et al., Marriage of 2D covalent–organic framework and 3D network as stable solar-thermal still for efficient solar steam generation. Small Meth. **5**(5), 2100036 (2021). <https://doi.org/10.1002/smtd.202100036>
33. S.Y. Zheng, J. Zhou, M. Si, S. Wang, F. Zhu et al., A molecularly engineered zwitterionic hydrogel with strengthened anti-polyelectrolyte effect: from high-rate solar desalination to efficient electricity generation. Adv. Funct. Mater. **33**(43), 2303272 (2023). <https://doi.org/10.1002/adfm.202303272>
